# Gravity Variations and Ground Deformations Resulting from Core Dynamics

**DOI:** 10.1007/s10712-021-09656-2

**Published:** 2021-09-30

**Authors:** Mathieu Dumberry, Mioara Mandea

**Affiliations:** 1 Department of Physics, University of Alberta, Edmonton, T6G 2E1 Canada; 2 Centre National d’Études Spatiales, 2 Place Maurice Quentin, 75039 Paris, France

**Keywords:** Gravity changes, Ground deformations, Core dynamics, Earth rotation

## Abstract

**Abstract:**

Fluid motion within the Earth’s liquid outer core leads to internal mass redistribution. This occurs through the advection of density anomalies within the volume of the liquid core and by deformation of the solid boundaries of the mantle and inner core which feature density contrasts. It also occurs through torques acting on the inner core reorienting its non-spherical shape. These in situ mass changes lead to global gravity variations, and global deformations (inducing additional gravity variations) occur in order to maintain the mechanical equilibrium of the whole Earth. Changes in Earth’s rotation vector (and thus of the global centrifugal potential) induced by core flows are an additional source of global deformations and associated gravity changes originating from core dynamics. Here, we review how each of these different core processes operates, how gravity changes and ground deformations from each could be reconstructed, as well as ways to estimate their amplitudes. Based on our current understanding of core dynamics, we show that, at spherical harmonic degree 2, core processes contribute to gravity variations and ground deformations that are approximately a factor 10 smaller than those observed and caused by dynamical processes within the fluid layers at the Earth’s surface. The larger the harmonic degree, the smaller is the contribution from the core. Extracting a signal of core origin requires the accurate removal of all contributions from surface processes, which remains a challenge.

**Article Highlights:**

- Dynamical processes in Earth's fluid core lead to global gravity variations and surface ground deformations
- We review how these processes operate, how signals of core origin can be reconstructed and estimate their amplitudes
- Core signals are a factor 10 smaller than the observed signals; extracting a signal of core origin remains a challenge

## Introduction

Planetary scale changes in Earth’s gravity field occur as a result of mass redistribution, either on its surface or deeper in its interior. Mass displacements at Earth’s surface are caused by a variety of processes in the atmosphere, ocean, hydrosphere and cryosphere, and also from mass exchanges between them. In situ mass variations also result from deformations of the ground surface and variations in mean sea level (and of any other density discontinuities), for instance from the elastic response to changes in mass load associated with the above processes, but also from tides and from pressure changes induced by winds and ocean currents. Other near surface processes such as seismic and volcanic deformations and deeper viscous mantle flows associated with convection and postglacial rebound also contribute to mass redistribution.

Until two decades ago, while the “static” (i.e. time invariable) part of Earth’s global gravity field was known with relatively good precision, our knowledge of its temporal changes was restricted to only that of its lowest spherical harmonic degrees. The situation drastically improved in 2002 with the launch of the GRACE mission, followed in 2018 by its successor GRACE-FO mission (e.g., Wahr 2015; Tapley et al. 2019). These missions have allowed us to measure the global temporal changes of Earth’s gravity with unprecedented spatiotemporal resolution and precision.

At a seasonal timescale and shorter, gravity variations are dominantly the result of mass redistribution and its associated loading at the Earth’s surface (e.g., Chen et al. 2016; Meyrath et al. 2017). Long-term variations, contributing to a secular trend over the few decades during which we have observations, are dominated by viscous mantle flows from convection and postglacial rebound (e.g., Tamisiea et al. 2007). The accelerating rate of melting of continental ice sheets and mountain glaciers induced by global warming over the past two decades, together with the elastic response of the solid Earth to this mass redistribution, is now imprinting a change in this secular trend, both in the degree 2, order 0 (elliptical) component of gravity (e.g., Nerem and Wahr 2011; Cheng and Ries 2018; Chao et al. 2020) but also in the degree 2, order 1 components and thereby inducing a displacement in the Earth’s rotation axis (or, for short, a polar motion) (e.g., Chen et al. 2013; Adhikari et al. 2018; Deng et al. 2021).

At interannual and decadal timescales, changes in terrestrial water storage and ocean mass appear to dominate the observed planetary scale gravity changes (Meyssignac et al. 2013; Adhikari and Ivins 2016; Sun et al. 2019). However, the fit between prediction and observation is not as good as for seasonal variations (Chen et al. 2016; Chao et al. 2020; Rosat et al. 2021). This is partly because models of these surface processes remain imperfect, especially those associated with terrestrial water storage. However, flows in Earth’s liquid core vary over interannual to decadal timescales (e.g., Holme 2015) and a part of the unexplained signal could be connected to these flows.

Dynamical processes in Earth’s fluid core can lead to a mass redistribution and hence to gravity variations in a few different ways. Density anomalies feed convective flows; their entrainment by core flows results in a time-dependent redistribution of mass (Dumberry 2010). A vertical motion in a stratified density layer generates an in situ density anomaly. Pressure changes induced by temporally varying core flows push and pull on the core-mantle boundary (CMB); elastic deformations resulting from these amounts to a mass displacement (Merriam 1988; Fang et al. 1996; Greff-Lefftz et al. 2004; Dumberry and Bloxham 2004). Because of the density contrast at the inner core boundary (ICB), a change in the orientation of the triaxial figure of the solid inner core likewise constitutes a mass displacement (Greiner-Mai et al. 2000; Greiner-Mai and Barthelmes 2001; Dumberry 2008a), and so would sudden or more gradual topography changes of the CMB, from the equivalent of landslides or avalanches, or from crystallization and dissolution (e.g., Mandea et al. 2015). Each of these processes involves global elastic deformations and also leads to ground deformations at the Earth’s surface.

How large a gravity signal can these core processes produce? And how can we construct predictions of such signals? In this article, we address these questions and review the different ways in which the core can generate gravity variations and ground deformations. The geometry and temporal variation of the large-scale core flows near the CMB can be reconstructed from the observed changes in the magnetic field (e.g., Holme 2015). This stems from the idea that in a good electrical conductor, such as the Earth’s core, magnetic field lines tend to be frozen-in to material particles. Predictions of the length of day changes ($\varDelta$LOD) from the reconstructed core flows agree well with the observed variations on decadal timescales (Jault et al. 1988; Jackson et al. 1993) but also for a signal with a period of approximately 6 year (Gillet et al. 2010, 2015). This demonstrates how dynamics within the core can be captured by geodetic observations. If a firm connection between core flows and gravity and/or ground deformations can be established, this opens the possibility of further constraining core dynamics from geodetic measurements. Moreover, it would ensure that a part of the observed geodetic signal is not wrongly attributed to processes in the atmosphere, oceans or continental hydrology.

A few recent studies have indeed suggested that specific observed geodetic signals may be connected to core dynamics. First, Mandea et al. (2012) showed that, after correcting for known surface contributions, the leading order global mode of temporal gravity variation recorded by satellites was well correlated with the leading order global mode of secular acceleration of the vertical component of the magnetic field. Their study covered a limited timespan between mid-2002 and mid-2010, but a recent follow-up study (Mandea et al. 2020) shows that the good correlation continues until the end of 2015. Two other studies (Ding and Chao 2018; Watkins et al. 2018) report a planetary scale $\sim 1$ mm amplitude signal in the surface displacement recorded by the Global Positioning System (GPS) network, each with a period of approximately 6 year. A 6-year signal is also observed in gravity observations (Chao and Yu 2020) and in polar motion (Chen et al. 2019). In each of these latter four studies, dynamical processes in the core were suggested to be at the origin of the observed signals, principally because of the demonstrated link between the axial angular momentum carried by core flows and $\varDelta$LOD changes at a 6-year period.

Detecting a clear contribution from the core in gravity and surface deformation observations would represent a new frontier for core dynamics. The recent studies listed above that have argued for a core origin of specific geodetic signals are briefly reviewed in the next section (Sect. 2). We then review the possible ways by which the core can induce variations in Sect. 3 and present estimate of the expected magnitude from each of the processes in Sect. 4. We conclude our article with a brief geophysical discussion in Sect. 5.

## Gravity Variations Possibly Connected to Core Dynamics

### Magnetic and Gravity Satellite Data: A Key to Unlocking Core Dynamics

Motion in Earth’s electrically conducting liquid outer core is driven by convection. This motion induces electrical currents generating and sustaining the Earth’s magnetic field. This highly nonlinear process is responsible for both the origin and the observed fluctuations of the geomagnetic field. Observation and analysis of the geomagnetic field variations provide the key to unlocking the time-dependent dynamics of the fluid core, as reviewed in Lesur et al. (2021).

The quality of global magnetic field observations has vastly improved over the past few decades with the advent of nearly continuous measurements from space on board satellites. The chief advantage of satellite measurements resides in their global coverage, removing possible geographical biases. After the MAGSAT mission (1979–1980), a wealth of new high-quality data followed from several near-Earth orbiting spacecrafts. To list a few: Ørsted, SAC-C, CHAMP, and recently Swarm, which involves a constellation of three satellites. The data obtained from Ørsted, CHAMP and Swarm in particular have proved very useful to build temporally varying global magnetic field models since 2000 (e.g., Olsen et al. 2009, 2014, 2016). Ørsted, launched in February of 1999, continues to this day to record scalar and vector magnetic field measurements and has served as a blueprint for subsequent satellite missions. CHAMP (Challenging Minisatellite Payload) mission^1^ was launched in 2000 and lasted until 2010. With its highly precise, multifunctional and complementary payload elements, as well as its orbital characteristics, CHAMP generated highly precise magnetic field measurements over its lifetime. The three Swarm^2^ satellites form a unique constellation dedicated to a detailed survey of the Earth’s magnetic field and its temporal evolution. In order to capture the magnetic field changes originating in the fluid core, the contributions from temporally varying induced magnetization in the lithosphere and from the currents in the ionosphere and magnetosphere have to be filtered out from the net magnetic field signal measured by satellites.

In parallel, satellite missions dedicated to the measurement of Earth’s gravity field in the past two decades have provided us with an unprecedented view of its global spatial and temporal variations. The GRACE (Gravity Recovery and Climate Experiment) twin-satellite mission^3^ orbited Earth from 2002–2017. A successor mission, GRACE-FO^4^ was launched in 2018, so there is a short gap in highly accurate gravity data. Precise tracking of the minute orbital fluctuations of satellites by the technique of Satellite Laser Ranging (SLR) also provides observations of the temporally changing gravity field. These include, in order of starting year of operation: Starlette (1975), LAGEOS-1 (LAser GEOdetic Satellite,1976), Ajisai (1986), LAGEOS-2 (1992), Stella (1993), and LARES (LAser RElativity Satellite, 2012).

The accuracy of the global temporal changes in gravity revealed by GRACE, GRACE-FO and SLR data opens the possibility of extracting a signature from core dynamics. The ways in which core processes can lead to temporal gravity variations are reviewed in Sect. 3, but the key point is that it must involve a redistribution of mass. A satellite is sensitive to the total gravity from all sources of mass variations, including those connected to tides, ocean circulation, atmospheric winds, pressure loading from the latter two, and terrestrial water storage. Hence, to extract a signature form the core in global gravity data, all surface contributions first have to be filtered out. This is a challenge, as our knowledge of these processes remains imperfect (e.g., Rosat et al. 2021).

### Global Correlation Between Magnetic and Gravity Signals

The challenge of identifying a signal of core origin in satellite gravity data was undertaken by Mandea et al. (2012). The aim of their study was to investigate spatio-temporal correlations between the global gravity and magnetic fields variations based on eight years of measurements provided by GRACE and CHAMP missions. CNES/GRGS geoid solutions based on GRACE data were used, from which gravity variations from the solid Earth, oceanic and atmospheric tides, and oceanic and atmospheric mass redistribution were already removed. Further efforts to remove any additional gravity contributions from processes at the Earth’s surface were applied in order to isolate a signal of core origin. Mandea et al. (2012) show that there is a good correlation between the remaining gravity signal and the secular acceleration of the radial component of the magnetic field in several geographical locations, in particular in a region centred around Africa. Furthermore, a singular value decomposition (SVD) was performed on both the global gravity field and the global radial secular acceleration time-series. Although the dominant mode for each time-series have different spatial structures, their inter-annual annual variations are well correlated (see their Fig. 4).

This analysis was recently revisited in a follow-up study by Mandea et al. (2020) over an extended time window, from January 2003 to December 2015, incorporating data from the GRACE-FO and Swarm missions. Several aspects of the data treatment and analysis are different from their earlier study. The gravity time-series were obtained from the pre-processed LEGOS V0.94 mean value model.^5^ To fill the gap from missing months, a cubic spline based on the annual and semi-annual cycles and the trend was applied. A Hamming window of 15 months was further applied to the time-series to minimize sub-annual signals and further efforts were taken to eliminate known gravity signals from surface processes. For the magnetic field, the CHAOS-6 model^6^ was used providing a very good continuous description of the radial component of the core field, its secular variation and secular acceleration.

**Fig. 1:**
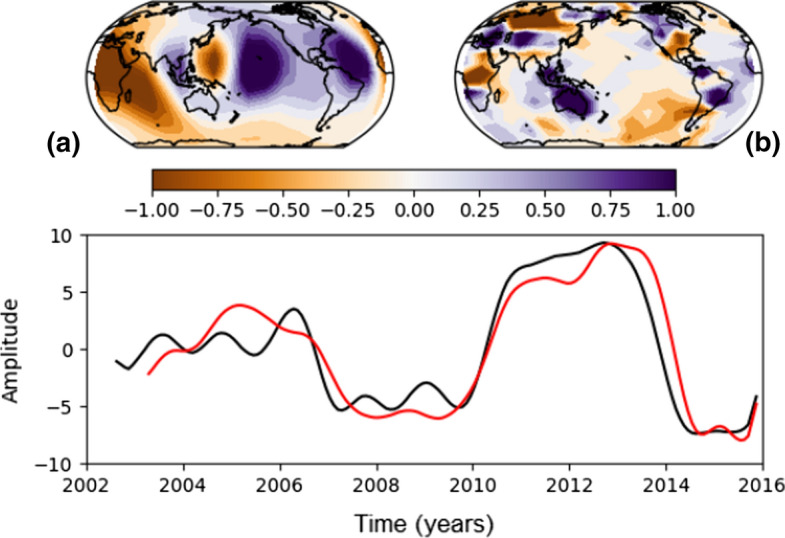
Common variability for virtual magnetic and gravity observatories series as obtained by applying a singular value decomposition technique. The spatial pattern of the dominant mode of the secular acceleration (SA) of the vertical downward magnetic field (upper panel a) and gravity field (upper panel b), and their temporal behaviour (lower panel, SA in red, in nT/$\hbox {yr}^2$, and gravity in black, in mm of equivalent water height (EWH))). As a note, 1 mm of EWH corresponds to a gravity change of 41.9 nGal; the amplitude of the gravity signal is of the order of 300 nGal. Figure reproduced from Mandea et al. (2020). (Note that the gravity and geomagnetic maps were incorrectly labelled in Mandea et al. (2020) and are corrected here.)

Both models were truncated at degree/order 8 in order to focus on the large spatial scale and represented on a global grid defined by boxes of 10$^\circ$ height in geographic latitude and 20$^\circ$ width in geographic longitude. This provides a well-distributed network of “virtual magnetic and gravity observatories” (VMGOs) located at the centre of each cell, each with its own time-series computed as monthly means. A singular value decomposition (SVD) was applied to both the gravity and magnetic time-series. Figure 1 shows the temporal and spatial variations of the dominant mode of the secular acceleration of the magnetic and of the filtered gravity field which, if all surface contributions were properly removed, would represent the signal from the core. The gravity and secular acceleration each feature a dominant sub-decadal signal that are very well correlated, confirming and extending the earlier results by Mandea et al. (2012). This hints that the gravity signal may indeed be originating in the core. However, the spatial structure of the dominant mode of gravity in Fig. 1b is different from that of the secular acceleration in Fig. 1a. Moreover, a cursory look at the spatial pattern of the gravity mode reveals that the largest changes are concentrated over continents. This indicates that a contribution from continental hydrology may still be contained in the remaining signal.

A mechanism is proposed in Mandea et al. (2015) for the common origin of both signals, based on a dissolution-crystallization process continuously reshaping the topography of the core-mantle boundary (CMB). In order to generate the observed gravity signal, they calculate that topography changes of the order of 30 cm (at harmonic degree $\sim$ 6) are required. Smaller topographic changes are actually needed if one were to take into account elastic deformations in the mantle (e.g., see Sect. 4.1). Mandea et al. (2015) argue that the changes in pressure associated with this topography change can generate geostrophic core flows and, in turn, radial flows that can produce the observed secular acceleration. However, whether such a mechanism can indeed explain the origin of the observed signals needs to be further investigated. First, when Mandea et al. (2015) scale their numerical experiments to Earth, they find that the typical timescale for reshaping the CMB is of the order of a billion years. They suggest that turbulent diffusivities could greatly accelerate the process, but this requires a reduction in timescale by a factor $10^8$. Second, the geostrophic flows involved in their process is $\sim 3 \times 10^{-3}$ m $\hbox {s}^{-1}$, an order of magnitude larger than the typical sub-decadal core flows inferred from the observed geomagnetic secular variation ($\sim 1\times 10^{-4}$ m $\hbox {s}^{-1}$, (e.g., Gillet et al. 2015). The amplitude of core flows that can be generated by CMB topography or pressure change is further discussed at the end of Sect. 4.1.

### Geodetic Variations of Harmonic Degree 2 with a 6-Year Period

Two recent studies (Ding and Chao 2018; Watkins et al. 2018) present evidence for a planetary scale (harmonic degree 2) vertical surface displacement of approximately 1 mm in amplitude caused by core processes. Watkins et al. (2018) show that the power spectrum of stacked Global Positioning System (GPS) data includes power at a period of 6 years. They fit a 6-year time-series curve to individual GPS stations data and invert for the non-hydrostatic pressure changes at the CMB consistent with those. From the degree 2, order 0 CMB pressure changes, and assuming tangential geostrophy (see Eq. 27), they calculate the changes in the azimuthal velocity of cylindrical surfaces aligned with the rotation axis inside the core. The changes in axial angular momentum computed from these shows reasonable agreement, both in phase and amplitude, with the observed 6-year $\varDelta$LOD, although the authors carefully point out that parts of their results may not be robust.

The study of Ding and Chao (2018) highlights the presence of a degree 2, order 2 signal in GPS observations, propagating westward and completing a half-rotation (and a full periodic cycle at a given point on the surface) in 5.9 year. This signal is found in both the vertical and horizontal ground displacement data. They further show that a similar signal is found in the magnetic field time-series recorded at surface observatories. The phase between the GPS signal, the magnetic signal and the 6-year $\varDelta$LOD shows remarkable agreement, suggesting that the signal is of core origin. In a follow-up study, Chao and Yu (2020) show that there is also a connected westward travelling signal in the degree 2, order 2 gravity field.

Complementing the picture, Chen et al. (2019) reports a 5.9 year signal in polar motion, the displacement of the position where the rotation axis intersects the Earth’s surface. If these are driven by mass redistribution, they capture the changing orientation of the rotation vector as it tracks the changing moment of inertia tensor of the planet. The latter are directly connected to the global gravity field of degree 2, order 1 (e.g., Gross 2015).

Hence, geodetic observations of degree 2 at all orders (0, 1 and 2) appear to include a 5.9 year signal. In all these studies, the authors argue that dynamics in the core is likely responsible for these signals. This is because of the 5.9 year period of the signals, and from the fact that the $\varDelta$LOD at a period of 5.9 year (e.g., Holme and de Viron 2013; Chao et al. 2014) can be explained by core-mantle angular momentum exchanges (Gillet et al. 2010, 2015). Furthermore, in each of these studies, efforts to remove the possible contribution from surface processes are documented.

However, the accuracy of some of these signals has been challenged. Rosat et al. (2021) reanalysed the GPS data and also found that the amplitude of the vertical displacement at a period of 5.9 year is of the order of 1 mm. When projected on spherical harmonic coefficients of degree 2, and after correcting for atmospheric and oceanic loading, the time-series signal of orders 0 and 1 both contain a 5.9 year signal, though with an amplitude closer to 0.5 mm. The signal of order 2, however, is much weaker ($\sim 0.18$ mm), with no clear indication of a systematic westward propagation. The power spectra of all coefficients of degree 2 contain peaks at similar frequencies than in the signal predicted by models of hydrological variations, and although the latter is weaker, Rosat et al. (2021) argue that this hints that the signal may be caused by surface processes. Rosat et al. (2021) also analysed gravity changes estimated by satellite laser ranging (SLR) and found that, although some correlations between the gravity and surface deformation signals of degree 2 are observed, important differences remain. This highlights the limited resolution of these global scale, interannual signals. Together with the large uncertainties associated with continental hydrology models, Rosat et al. (2021) conclude that it is not possible at present to firmly establish that the gravity variations and ground deformations at a period of 6 year originate from a dynamical process in Earth’s core.

## Theory: Changes in Gravity and Surface Deformation from Core Dynamics

**Fig. 2:**
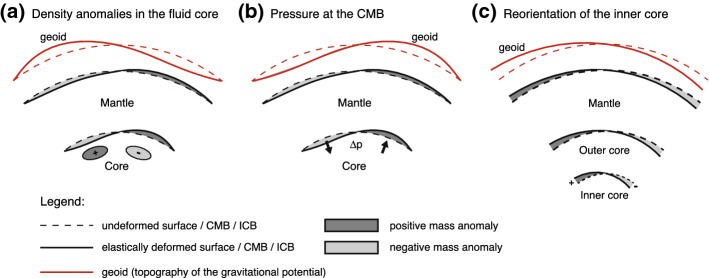
Geoid (topography of the gravitational potential) variations at the Earth’s surface (red) caused by mass displacements originating in the core. **a** Temporal displacements of density anomalies (“+”, “−”) connected with core dynamics lead to variations in the geoid. The latter include the contribution from global elastic deformations which occur in response to the altered internal gravity field. **b** Temporal changes in the horizontal pressure gradient alter the local normal surface force (arrows) on the CMB, leading to global elastic deformations and to a change in the geoid. **c** An axial or equatorial re-orientation of the triaxial inner core creates a degree 2 mass anomaly at the ICB (“+”, “−”) which leads to a variation in degree 2 of the geoid. As in (**a**), the latter includes the contribution from global elastic deformations which occur in response to the altered internal gravity field. Note that in all three panels, the deflection of the radial gradient of density within the mantle and core also contributes to density perturbations, but for ease of illustration only that resulting from the deflection of the CMB and the external surface are shown. Figure modified from Dumberry (2010)

Mass redistribution within the Earth’s core results in global gravity changes. The simplest way this occurs is through the displacement of density anomalies by core flows (Fig. 2a) (Dumberry 2010). To maintain mechanical equilibrium, the perturbed global gravitational force must be balanced by an adjustment in the internal stress field. This is achieved through global elastic deformations, which entrain a secondary density perturbation further contributing to the change in gravity. Density anomalies within the volume of the core can also result from vertical motion in a stably stratified layer; the presence of such a layer has been suggested at the top of the core (e.g., Tanaka 2007; Helffrich and Kaneshima 2010; Buffett 2014).

There is a large density contrast between the core and the mantle at the CMB, and an additional source of mass variation results from the deformation of the CMB induced by changes in lateral gradients of the non-hydrostatic pressure field. The change in the local normal surface force that this creates alters the global mechanical equilibrium, leading to global elastic deformations and, consequently, to changes in gravity (Fig. 2b) (Merriam 1988; Fang et al. 1996; Greff-Lefftz et al. 2004; Dumberry and Bloxham 2004). The same process occurs at the inner core boundary (ICB).

The evolving dynamics in the fluid core above the ICB lead to temporally varying torques acting on the inner core. The latter adjusts by changing its instantaneous orientation with respect to the mantle. Because of the density contrast at the ICB, and because the ICB is not spherically symmetric, a rotation of the inner core amounts to a mass displacement and thus to a gravity change (Fig. 2c). This can occur both as a result of an axial rotation (connected to the longitudinal variations in ICB topography (e.g., Buffett 1996a; Mound and Buffett 2003)) or an equatorial rotation (caused by the oblate elliptical figure of the inner core (Greiner-Mai et al. 2000; Greiner-Mai and Barthelmes 2001; Dumberry 2008a).

An additional indirect way by which core dynamics can lead to changes in gravity is through the changes in the rotation vector of the solid Earth that they induce. For instance, axial torques between the core and mantle lead to $\varDelta$LOD or, in other words, a change in the amplitude of rotation of the mantle. Likewise, equatorial torques between the core and the mantle lead to change in the orientation of the rotation axis with respect to the mantle (a polar motion). Changes in the amplitude and orientation of the rotation vector modify the centrifugal potential in the mantle, leading to global elastic deformations primarily of harmonic degree 2, and hence to a change in the degree 2 gravity field.

Changes in the gravity field, caused by a mass redistribution in the core or anywhere else within or outside Earth, are expressed as changes in the gravitational potential. The usual convention in geodesy is to express the gravitational potential $V(r,\theta ,\phi )$ at a radius *r* above the Earth’s surface and at co-latitude $\theta$ and longitude $\phi$ by a surface spherical harmonic expansion,

1 $$\begin{aligned} V(r,\theta ,\phi )= - \frac{G M}{r} \left[ 1 + \sum _{l=1}^{\infty } \sum _{m=0}^l \left( \frac{R}{r}\right) ^l \left( C_{lm} \cos m\phi + S_{lm} \sin m\phi \right) {\bar{P}}_l^m(\cos \theta ) \right] \, , \end{aligned}$$

where *G* is the gravitational constant, *M* is the mass of the Earth and *R* its mean spherical radius; their values are given in Table 1. The coefficients $C_{lm}$ and $S_{lm}$ are known as Stokes coefficients, and the associated Legendre polynomials ${\bar{P}}_l^m(\cos \theta )$ are normalized such that

2 $$\begin{aligned} \int \left[ {\bar{P}}_l^m(\cos \theta ) \cos m\phi \right] ^2 d\varOmega = \int \left[ {\bar{P}}_l^m(\cos \theta ) \sin m\phi \right] ^2 d\varOmega ={4 \pi } \, , \end{aligned}$$

where the integration is over a unit sphere. They are related to the regular (unnormalized) associated Legendre polynomials ${P}_l^m(\cos \theta )$ by

3 $$\begin{aligned} {\bar{P}}_l^m(\cos \theta ) = \left[ (2-\delta _{m,0}) (2l+1) \frac{(l-m)!}{(l+m)!} \right] ^{1/2} {P}_l^m(\cos \theta ) \, , \end{aligned}$$

where $\delta _{m,0}$ is the Kronecker delta function.

The radial component of the gravity field is $\mathbf{g_r} = - g_o {\hat{\mathbf{r}}}$, where the downward pointing scalar gravity field $g_o = \frac{\partial V}{\partial r}$ is given by

4 $$\begin{aligned} g_o(r,\theta ,\phi ) = \frac{G M}{r^2} \left[ 1 + \sum _{l=1}^{\infty } \sum _{m=0}^l (l+1) \left( \frac{R}{r}\right) ^l \left( C_{lm} \cos m\phi + S_{lm} \sin m\phi \right) {\bar{P}}_l^m(\cos \theta ) \right] \, . \end{aligned}$$

In both Eqs. (1) and (4), the first term of the expansion on the right-hand side corresponds to the spherically symmetric part of the gravity field, whereas the summation term captures its non-spherical part. Due to the latter, surfaces of constant gravitational potential are no longer spherical. Its topography at the Earth’s surface is referred to as the geoid.

Dynamical core processes contribute to a quasi-steady part of the non-spherical gravity field. However, at the Earth’s surface, this contribution is of the order of a few hundred nanoGals (Greff-Lefftz et al. 2004) and is very small compared to that from mass anomalies in the crust and mantle—which are of the order of 10 to 100 milliGals (e.g., Förste et al. 2008)—and cannot be unambiguously identified. A better prospect to detect a core signature in the gravity field is through its temporal changes. Specifically, we will be investigating the changes in time of the Stokes coefficients and gravity field, which we denote by $\varDelta C_{lm}$ and $\varDelta S_{lm}$.

Gravity changes are alternately reported in terms of the amplitude of $\varDelta C_{lm}$ and $\varDelta S_{lm}$, or directly in gravity units. The connection between the two is through Eq. (4), where we can write ${\bar{g}}=GM/R^2=9.82$ m $\hbox {s}^{-2}$ for the mean spherical gravitational acceleration at the Earth’s surface. Expressed in units of nanoGals (nGal), where 1 nGal = $10^{-11}$ m $\hbox {s}^{-2}$, a given $\varDelta C_{lm}$ represents a gravity change of ${\bar{g}}\cdot 10^{11} \cdot (l+1) \cdot \varDelta C_{lm}$ multiplied by the surface spherical harmonic function ${\bar{P}}_l^m (\cos \theta ) \cos m\phi$. For instance, the maximum amplitude of ${\bar{P}}_2^2$ is $\pm \sqrt{15/4}$, so $\varDelta C_{22}=10^{-11}$ corresponds to a gravity amplitude of $9.82 \cdot 3 \cdot \sqrt{15/4} = 57.0$ nGal. Gravity changes are sometimes reported in terms of equivalent changes in geoid height, which is obtained by multiplying the change in Stokes coefficients by Earth’s radius and by its surface spherical harmonic function; continuing the example above, $\varDelta C_{22}=10^{-11}$ corresponds to a geoid height change of $\sqrt{15/4} \cdot R \cdot \varDelta C_{22} = 0.123$ mm. Finally, gravity variations at geographic locations are also sometimes reported in terms of equivalent water height (EWH), which is the gravity equal to $2\pi \cdot 10^{11} G \rho _w h$ (in units of nGal) produced by an equivalent layer of thickness *h* of water with density $\rho _w = 10^3$ kg $\hbox {m}^{-3}$. A 1 mm EWH change is equal to 41.9 nGal, so the gravity anomaly of 57 nGal in our above example corresponds to an EWH of 1.36 mm.

Because all sources of gravity variations identified above involve global elastic deformations, they are accompanied by vertical and lateral displacements at the Earth’s surface. These offer a separate and complementary way to detect a geodetic signal possibly originating from the core. Let us use an expansion for the temporal variations of the vertical displacements $\varDelta U$ at the Earth’s surface (radius *R*) and $\varDelta W$ at the CMB (radius $r_f$) in the same form as that for the gravitational potential,

5a $$\begin{aligned} \varDelta U(R,\theta ,\phi )&= \sum _{l=1}^{\infty } \sum _{m=0}^l \left( \varDelta U_{lm}^c \cos m\phi + \varDelta U_{lm}^s \sin m\phi \right) {\bar{P}}_l^m(\cos \theta ) \, , \end{aligned}$$

5b $$\begin{aligned} \varDelta W(r_f,\theta ,\phi )&= \sum _{l=1}^{\infty } \sum _{m=0}^l \left( \varDelta W_{lm}^c \cos m\phi + \varDelta W_{lm}^s \sin m\phi \right) {\bar{P}}_l^m(\cos \theta ) \, . \end{aligned}$$

Whereas the Stokes coefficients $\varDelta C_{lm}$, $\varDelta S_{lm}$ are dimensionless, the vertical displacement coefficients $\varDelta U_{lm}^{c,s}$ and $\varDelta W_{lm}^{c,s}$ have units of distance.

Our task is then to connect and quantify how different core processes lead to temporal variations in the Stokes coefficients, $\varDelta C_{lm}$, $\varDelta S_{lm}$ and vertical displacement coefficients $\varDelta U_{lm}^{c,s}$, $\varDelta W_{lm}^{c,s}$; the formalism to do so is presented in the remainder of this section.

**Table 1:** Earth parameters used in calculations

Parameter	value
Gravitational constant	$G=6.674 \times 10^{-11}$ $\hbox {m}^3$ $\hbox {kg}^{-1}$ $\hbox {s}^{-2}$
Mass of the Earth	$M=5.972 \times 10^{24}$ kg
Radius of Earth	$R = 6.371 \times 10^6$ m
Radius of the core	$r_f = 3.480 \times 10^6$ m
Radius of the inner core	$r_s = 1.222 \times 10^6$ m
Mean Earth density	${\bar{\rho }} = 5 515$ kg $\hbox {m}^{-3}$
Density of core at CMB	$\rho _f = 9 903$ kg $\hbox {m}^{-3}$
Density jump at the ICB	$\varDelta \rho _\mathrm{icb} = 600$ kg $\hbox {m}^{-3}$
Gravitational acceleration at surface	${\bar{g}} = 9.82$ m $\hbox {s}^{-2}$
Rotation frequency	$\varOmega _o =7.292 \times 10^{-5}$ $\hbox {s}^{-1}$
Axial moment of inertia of the mantle	$C_m=7.129 \times 10^{37}$ kg $\hbox {m}^2$
Axial moment of inertia of the core	$C_c=0.908 \times 10^{37}$ kg $\hbox {m}^2$

### Density Anomalies in the Fluid Core

The equilibrium hydrostatic density within the fluid core varies as a function of radius. It also includes an elliptical component and additional smaller amplitude variations with latitude and longitude induced by mass anomalies in the mantle. We can denote this background hydrostatic density as $\rho _o(r,\theta ,\phi )$. A density perturbation $\varDelta \rho (r,\theta ,\phi )$ at a radius *r* with respect to $\rho _o(r,\theta ,\phi )$ within the fluid core results in a global change in the gravitational potential. Let us expand $\varDelta \rho (r,\theta ,\phi )$ as a sum of surface spherical harmonics with coefficients ${\rho }_{lm}^c(r)$ and ${\rho }_{lm}^s(r)$,

6 $$\begin{aligned} \varDelta \rho (r,\theta ,\phi ) = \sum _{l=1}^{\infty } \sum _{m=0}^l \left[ {\rho }_{lm}^c(r) \, \cos m\phi + {\rho }_{lm}^s(r) \, \sin m\phi \right] {\hat{P}}_l^m(\cos \theta ) \, , \end{aligned}$$

where the associated Legendre polynomials ${\hat{P}}_l^m(\cos \theta )$ follow a Gauss–Schmidt normalization

7 $$\begin{aligned} \int \left[ {\hat{P}}_l^m(\cos \theta ) \cos m\phi \right] ^2 d\varOmega = \int \left[ {\hat{P}}_l^m(\cos \theta ) \sin m\phi \right] ^2 d\varOmega =\frac{4 \pi }{2l+1} \, . \end{aligned}$$

The latter normalization is more conventionally used in geomagnetism and core dynamics and this is why we have adopted it here. The Gauss–Schmidt normalized ${\hat{P}}_l^m(\cos \theta )$ are connected to the ${\bar{P}}_l^m(\cos \theta )$ used in geodesy by ${\hat{P}}_l^m(\cos \theta ) = {\bar{P}}_l^m(\cos \theta )/ \sqrt{2l+1}$, and to the regular (unnormalized) associated Legendre polynomials ${P}_l^m(\cos \theta )$ by

8 $$\begin{aligned} {\hat{P}}_l^m(\cos \theta ) = \left[ (2-\delta _{m,0}) \frac{(l-m)!}{(l+m)!} \right] ^{1/2} {P}_l^m(\cos \theta ) \, . \end{aligned}$$

The change in gravitational potential at the surface (radius *R*) depends on the density variations integrated within the whole of the fluid core, from the ICB (radius $r_s$) to the CMB (radius $r_f$). The change in Stokes coefficients caused by temporal changes in density coefficients ${\rho }_{lm}^c(r)$ and ${\rho }_{lm}^s(r)$ is (Dumberry 2010)

9a $$\begin{aligned} \varDelta C_{l m}&= \frac{4 \pi }{(2l+1)^{3/2}} \frac{1}{M R^{l}} \int _{r_s}^{r_f} \rho _{lm}^c(r) \, \left[ 1+\kappa _l(r) \right] r^{l+2} \text{ d }r \, , \end{aligned}$$

9b $$\begin{aligned} \varDelta S_{l m}&= \frac{4 \pi }{(2l+1)^{3/2}} \frac{1}{M R^{l}} \int _{r_s}^{r_f} \rho _{lm}^s(r) \, \left[ 1+\kappa _l(r)\right] r^{l+2} \text{ d }r \, . \end{aligned}$$

The functions $\kappa _l(r)$ characterize the added contribution associated with the global elastic deformations that accompany a local density change of harmonic degree *l* located at radius *r*. Their numerical values depend on how the hydrostatic density and elastic moduli vary as a function of radius inside the Earth.

**Fig. 3:**
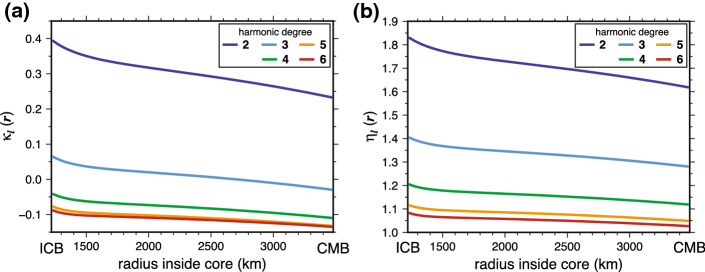
Elastic parameters **a** $\kappa _l(r)$ and **b** $\eta _l(r)$ as a function of the radius in the fluid core at which a surface mass density anomaly is applied and for different harmonic degree *l*. Calculations are based on PREM (Dziewonski and Anderson 1981)

The functions $\kappa _l(r)$ were computed by Dumberry (2010) for PREM Earth model (Dziewonski and Anderson 1981). Their computation involves solving a set of elastic-gravitational equations that capture how the mechanical equilibrium between surface tractions and gravitational forces in the whole Earth is altered in the presence of an imposed internal mass anomaly. It follows a well-established procedure (e.g., Alterman et al. 1959), one which has also been used to compute the elastic deformations connected to mass anomalies located in the mantle (e.g., Richards and Hager 1984; Defraigne et al. 1996). Elastic deformations at harmonic degree $l=1$ involve a slightly modified system of equations compared to those for $l \ge 2$, notably to ensure conservation of the centre of mass (e.g., Greff-Lefftz and Legros 1997). We concentrate on $l \ge 2$ and Fig. 3a shows how the functions $\kappa _l(r)$ vary as a function of the radius *r* at which the density anomaly occurs and for $l=2$ to $l=6$. For $l=2$, elastic deformations increase the amplitude of the gravitational potential at the surface by approximately 30%. For $l > 2$, elastic deformations contribute to a change of approximately 10% or less compared to a rigid Earth. We note that $\kappa _l(r)$ can be both positive or negative: whether elastic deformations contribute to an increase or decrease in the resulting potential at the surface is a non-trivial function of the Earth model and of the radial location and horizontal extend of the density anomaly.

The coefficients of vertical displacement at the Earth’s surface that results from density anomalies in the core are expressed as

10a $$\begin{aligned} \varDelta U_{lm}^c&= \frac{4 \pi }{(2l+1)^{3/2}} \frac{1}{M R^{l-1}} \int _{r_s}^{r_f} \rho _{lm}^c(r) \, \eta _l(r) \, r^{l+2} \text{ d}r \, , \end{aligned}$$

10b $$\begin{aligned} \varDelta U_{lm}^s&= \frac{4 \pi }{(2l+1)^{3/2}} \frac{1}{M R^{l-1}} \int _{r_s}^{r_f} \rho _{lm}^s(r) \, \, \eta _l(r)\, r^{l+2} \text{d}r \, . \end{aligned}$$

The functions $\eta _l(r)$ capture the ground deformation at the Earth’s surface associated with global elastic deformations that accompany a local density change of harmonic degree *l* located at radius *r*. They are calculated following the method given in Dumberry (2010) and are shown in Fig. 3b.

Note that according to Eqs. (9) and (10), for a mass anomaly located near the CMB, both the Stokes coefficients $\varDelta C_{l m}$, $\varDelta S_{l m}$ and vertical displacement coefficients $\varDelta U_{lm}^c$, $\varDelta U_{lm}^s$ vary as $(r_f/R)^l(2l+1)^{-3/2} \approx 2^{-l}(2l+1)^{-3/2}$ with harmonic degree *l*. A mass anomaly of the same amplitude but higher harmonic degree results in a smaller signal at the Earth’s surface.

### Pressure Changes at the Core-Mantle Boundary

Pressure anomalies at the CMB connected to core dynamics lead to changes in the normal force applied on the CMB. These, in turn, lead to global elastic deformations and to an associated change in gravity at the surface. Let us expand the non-hydrostatic pressure anomalies $\varDelta p (\theta ,\phi )$ at the CMB as a sum of surface spherical harmonics using Gauss–Schmidt normalized associated Legendre polynomials,

11 $$\begin{aligned} \varDelta p (\theta ,\phi ) = \sum _{l=1}^{\infty } \sum _{m=0}^l \left[ p_{lm}^c \cos m\phi + p_{lm}^s \sin m\phi \right] {\hat{P}}_l^m(\cos \theta ) \, . \end{aligned}$$

The connection between the pressure coefficients $p_{lm}^c$ and $p_{lm}^s$ and the Stokes coefficients is given by (e.g., Eqs. (14, 15) of Dumberry 2010),

12 $$\begin{aligned} \varDelta C_{lm} = \frac{{\bar{k}}_{l}}{\sqrt{2l+1}} \frac{R}{G M {\bar{\rho }}} \, p_{lm}^c \, ,\quad \varDelta S_{lm} = \frac{{\bar{k}}_{l}}{\sqrt{2l+1}} \frac{R}{G M {\bar{\rho }}} \, p_{lm}^s \, , \end{aligned}$$

where ${\bar{\rho }}$ is the mean density of the Earth and ${\bar{k}}_l$ are Love numbers. The latter are coefficients that capture how a unit pressure change at the CMB of harmonic degree *l* translates to a gravitational potential at the surface as a result of global elastic deformations. Their numerical values were computed in Dumberry and Bloxham (2004) and Greff-Lefftz et al. (2004) for $l \ge 2$ based on PREM and are given in Table 2.

The coefficients of the vertical displacement at the surface ($\varDelta U_{lm}^{c,s}$) and CMB ($\varDelta W_{lm}^{c,s}$) are connected to the CMB pressure coefficients by (e.g., Eq. 50 of Dumberry and Bloxham 2004, though note that we use here a different spherical harmonic normalization for the surface displacement)

13 $$\begin{aligned} {\varDelta U}_{lm}^{c,s} = \frac{{\bar{h}}_{l}}{\sqrt{2l+1}} \frac{1}{{\bar{\rho }} {\bar{g}}} \, p_{lm}^{c,s} \, , \quad {\varDelta W}_{lm}^{c,s} = \frac{{\bar{h}}^c_{l}}{\sqrt{2l+1}} \frac{1}{{\bar{\rho }} {\bar{g}}} \, p_{lm}^{c,s} \, , \end{aligned}$$

where ${\bar{g}}=9.82$ m $\hbox {s}^{-2}$ is the gravitational acceleration at the Earth’s surface and ${\bar{h}}_l$ and ${\bar{h}}^c_l$ are vertical displacement Love numbers. The numerical values of ${\bar{h}}_l$ were computed in Dumberry and Bloxham (2004) and Greff-Lefftz et al. (2004) and those of ${\bar{h}}^c_l$ are computed here following the same methodology; they are given in Table 2.

**Fig. 4:**
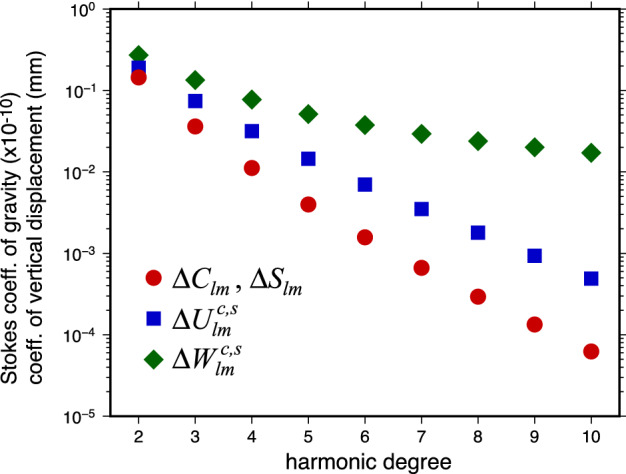
Stokes coefficients ($\varDelta C_{lm}$, $\varDelta S_{lm}$) from Eq. (12) and coefficients of vertical displacements at the surface (${\varDelta U}_{lm}^{c,s}$) and CMB (${\varDelta W}_{lm}^{c,s}$) from Eq. (13) as a function of harmonic degree *l* for an assumed CMB pressure coefficient $p_{lm}^{c,s}=100$ Pa

An important point to notice is that both ${\bar{k}}_l$ and ${\bar{h}}_l$ decrease rapidly with increasing harmonic degree; ${\bar{k}}_l$ decreases by a factor of approximately 1000 and ${\bar{h}}_l$ by a factor of approximately 100 between degrees 2 and 10. A pressure change at the CMB of the same amplitude but higher harmonic degree results in smaller gravity and ground deformation signals at the Earth’s surface. Typical CMB pressure changes generated by core flows are of the order of 100 Pa. A graphical representation of the decrease in the amplitude of the Stokes coefficients $\varDelta C_{lm}$, $\varDelta S_{lm}$ and in the coefficients of vertical displacements ${\varDelta U}_{lm}^{c,s}$ and ${\varDelta W}_{lm}^{c,s}$ as a function of harmonic degree *l* is shown in Fig. 4, assuming a pressure coefficient $p_{lm}^{c,s}=100$ Pa.

**Table 2:** Love numbers associated with pressure changes at the CMB

Degree	${\bar{k}}_l$	${\bar{h}}_l$	${\bar{h}}^c_l$
2	$1.116 \times 10^{-1}$	$2.302 \times 10^{-1}$	$3.294 \times 10^{-1}$
3	$3.304 \times 10^{-2}$	$1.064 \times 10^{-1}$	$1.923 \times 10^{-1}$
4	$1.156 \times 10^{-2}$	$5.135\times 10^{-2}$	$1.258 \times 10^{-1}$
5	$4.560 \times 10^{-3}$	$2.598\times 10^{-2}$	$9.210 \times 10^{-2}$
6	$1.957 \times 10^{-3}$	$1.366\times 10^{-2}$	$7.330 \times 10^{-2}$
7	$8.873 \times 10^{-4}$	$7.357\times 10^{-3}$	$6.153 \times 10^{-2}$
8	$4.171 \times 10^{-4}$	$4.013 \times 10^{-3}$	$5.339 \times 10^{-2}$
9	$2.010 \times 10^{-4}$	$2.204 \times 10^{-3}$	$4.734\times 10^{-2}$
10	$9.856\times 10^{-5}$	$1.215 \times 10^{-3}$	$4.261 \times 10^{-2}$

Calculations are based on PREM (Dziewonski and Anderson 1981)

### Core Induced Changes in Earth’s Rotation

Temporally varying axial torques exerted by the core on the mantle results in a change in the rotation rate of the latter. This change in mantle rotation, which we denote by $\delta \varOmega _m$, modifies the centrifugal potential within the mantle. This leads to global elastic deformations—and thus to gravity variations—of degree 2, order 0. Hence, time-dependent core flows that induce an exchange of angular momentum between the core and mantle can, indirectly, also lead to gravity changes. Likewise, an axial torque on the inner core changes its rotation rate by $\delta \varOmega _{ic}$, leading to a centrifugal potential change within the inner core which is also accompanied by global elastic deformations and a change in gravity.

Let us denote by $Z_2^{(m)}$ and $Z_2^{(ic)}$ the centrifugal potential changes in the mantle and inner core, respectively. They are connected to $\delta \varOmega _m$ and $\delta \varOmega _{ic}$ by (e.g., Dumberry and Bloxham 2004)

14 $$\begin{aligned} Z_2^{(m)} = \frac{2}{3} R^2 \varOmega _o \, \delta \varOmega _m \, , \quad Z_2^{(ic)} = \frac{2}{3} R^2 \varOmega _o \, \delta \varOmega _{ic} \, , \end{aligned}$$

where $\varOmega _o$ is the Earth’s rotation rate. The change in the degree 2, order 0 gravity is

15 $$\begin{aligned} \varDelta C_{20}&= - \frac{1}{\sqrt{5}} \frac{R}{GM} \left( k_2^{(m)} Z_2^{(m)} + k_2^{(ic)} Z_2^{(ic)} \right) \\ \nonumber&= - \frac{1}{\sqrt{5}} \frac{2}{3} \frac{R^3 \varOmega _o}{GM} \left( k_2^{(m)} \delta \varOmega _m + k_2^{(ic)} \delta \varOmega _{ic} \right) \, , \end{aligned}$$

where the Love numbers $k_2^{(m)}=0.2345$ and $k_2^{(ic)}=1.47 \times 10^{-6}$ were calculated in Dumberry and Bloxham (2004). Because $k_2^{(ic)} \ll k_2^{(m)}$, the contribution to a change in inner core rotation rate to $\varDelta C_{20}$ is negligible. Furthermore, assuming time-dependent zonal core flows that are invariant in the direction of the rotation axis, the change in angular velocity of the core $\delta \varOmega _c$ can be directly related to the degree 2, order 0 coefficient of pressure at the CMB (see Dumberry and Bloxham 2004),

16 $$\begin{aligned} p_{20}^c = - \frac{2}{3} \rho _f \varOmega _o r_f^2 \, \delta \varOmega _c \, , \end{aligned}$$

where $\rho _f$ is the density of the fluid core at the CMB. By conservation of angular momentum, $\delta \varOmega _m$ and $\delta \varOmega _c$ are connected by $C_m \delta \varOmega _m = - C_c \delta \varOmega _c$, where $C_m$ and $C_c$ are the axial moment of inertia of the mantle and core, respectively. Hence, we can express $\delta \varOmega _m$ in terms of $p_{20}^c$ by

17 $$\begin{aligned} \delta \varOmega _m = \frac{3}{2} \frac{C_c}{C_m} \frac{p_{20}^c}{\rho _f \varOmega _o r_f^2} \, , \end{aligned}$$

and we can write

18 $$\begin{aligned} \varDelta C_{20} = - \frac{k_2^{(m)}}{\sqrt{5}} \frac{R}{GM \rho _f} \frac{C_c}{C_m} \left( \frac{R}{r_f}\right) ^2 p_{20}^c \, . \end{aligned}$$

The vertical displacement of degree 2, order 0 at the Earth’s surface resulting from changes in the rotation rates of the mantle and inner core can be written as

19 $$\begin{aligned} \varDelta U_{20}^c = - \frac{1}{\sqrt{5}} \frac{1}{{\bar{g}}} \left( h_2^{(m)} Z_2^{(m)} + h_2^{(ic)} Z_2^{(ic)} \right) = - \frac{1}{\sqrt{5}} \frac{2}{3} \frac{R^2 \varOmega _o}{{\bar{g}}} \left( h_2^{(m)} \delta \varOmega _m + h_2^{(ic)} \delta \varOmega _{ic} \right) \, , \end{aligned}$$

where the Love numbers $h_2^{(m)}=0.4769$ and $h_2^{(ic)}=1.29 \times 10^{-6}$ were calculated in Dumberry and Bloxham (2004). Proceeding as above and neglecting the contribution from the inner core,

20 $$\begin{aligned} \varDelta U_{20}^c = - \frac{h_2^{(m)}}{\sqrt{5}} \frac{1}{{\bar{g}} \rho _f} \frac{C_c}{C_m} \left( \frac{R}{r_f}\right) ^2 p_{20}^c \, . \end{aligned}$$

An axially invariant zonal core flow generating a pressure change of degree 2, order 0 at the CMB produces then a change in $\varDelta C_{20}$ and $\varDelta U_{20}^c$ by elastic deformations from two different origins: from the gradient in the normal force pushing on the CMB (Eqs. 12 and 13) and from the change in the mantle rotation rate (Eqs. 18 and 20). These forces act opposite one another, and the net effect of a change in $p_{20}^c$ can be written as

21a $$\begin{aligned} \varDelta C_{20} = \frac{{\bar{k}}_{2}}{\sqrt{5}} \frac{R}{G M {\bar{\rho }}} \left( 1 - \zeta _k \right) p_{20}^c \, , \quad \varDelta U_{20}^c = \frac{{\bar{h}}_{2}}{\sqrt{5}} \frac{1}{{\bar{g}} {\bar{\rho }}} \left( 1 - \zeta _h \right) p_{20}^c \, , \end{aligned}$$

where

21b $$\begin{aligned} \zeta _k = \frac{k_2^{(m)}}{{\bar{k}}_2} \frac{{\bar{\rho }}}{\rho _f} \frac{C_c}{C_m} \left( \frac{R}{r_f}\right) ^2 \, , \quad \zeta _h = \frac{h_2^{(m)}}{{\bar{h}}_2} \frac{{\bar{\rho }}}{\rho _f} \frac{C_c}{C_m} \left( \frac{R}{r_f}\right) ^2 \, . \end{aligned}$$

When taking the parameter values from Table 1, $\zeta _k =0.4995$ and $\zeta _h =0.4925$.

### Reorientation of the Inner Core

Assuming hydrostatic equilibrium, the shape of the inner core is an oblate ellipsoid of revolution. The geometric ellipticity, or equivalently the flattening, of the ICB is $\epsilon _s = (r_s^{eq}- r_s^p)/r_s \approx 2.5 \times 10^{-3}$ (e.g., Mathews et al. 1991), where $r_s^{eq}$, $r_s^{p}$ and $r_s$ denote, respectively, the equatorial, polar and mean radii at the ICB. With $r_s=1222$ km, the difference between the equatorial and polar radii is approximately 3 km. Because of the density contrast at the ICB of $\varDelta \rho _{icb} \approx 600$ kg $\hbox {m}^{-3}$ (e.g., Gubbins et al. 2008), the elliptical shape of the ICB represents the largest non-spherical density anomaly within the whole of the core. Additional density anomalies within the inner core can possibly exist, either frozen-in or feeding convective motion. The latter would also induce a dynamical topography of the ICB, resulting in an additional density anomaly. The amplitude and wavelength of such a topography, whether frozen-in or dynamically maintained, are at present unknown. If the inner core is not convecting and if its density is close to uniform, the ICB should coincide with an equipotential surface at hydrostatic equilibrium. In addition to its flattening, its shape must then match the imposed gravitational potential from other non-spherical mass anomalies in the mantle (e.g., Buffett 1996a). The largest of those is a degree 2, order 2 anomaly; the topography of the equipotential surface (the geoid) at the ICB, $h_{22}^{s}$, is connected to the topography of the geoid at the CMB, $h_{22}^{f}$, through $h_{22}^{s} = (r_s/r_f) h_{22}^{f}$. Taking $h_{22}^{f} \approx 50$ m (e.g., Čadek and Fleitout 2006), this gives $h_{22}^{s} \approx 18$ m.

The evolving dynamics in the fluid core near the ICB generates torques on the inner core which alter its orientation with respect to the mantle. A torque directed along an equatorial direction leads to a meridional rotation of the flattened ICB producing a mass redistribution and a gravity change of degree 2, order 1. An axial torque leads to a longitudinal displacement of the degree 2, order 2 ICB topography, producing a gravity change of the same degree and order. The ICB topography invariably includes features of higher harmonic degrees, and the inner core density is likely not perfectly uniform. A change in inner core orientation should then also lead to gravity changes of higher degrees. However, since the gravity anomaly at the Earth’s surface caused by an ICB topography of degree *l* involves a factor $(r_s/R)^l$, the degree 2 dominates the signal at the surface and we focus our attention on this signal.

**Fig. 5:**
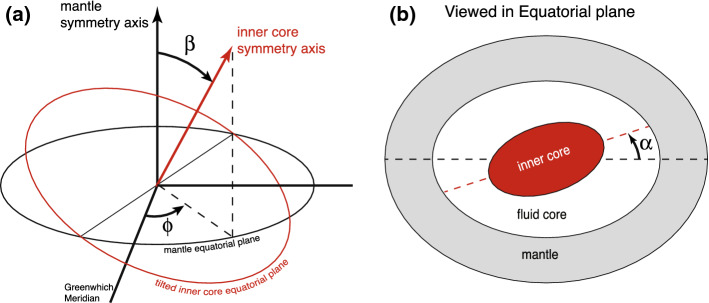
**a** A tilt by an angle $\beta$ of the symmetry axis of the inner core in a longitudinal direction $\phi$ with respect to the mantle frame. **b** An axial rotation of the long equatorial axis of the inner core (dashed red line) by an azimuthal angle $\alpha$ with respect to the long equatorial axis of the mantle (dashed black line) as viewed in the equatorial plane. Ellipticities are not drawn to scale

Assuming an inner core with uniform density and a density contrast at the ICB of $\varDelta \rho _{icb}$, a tilt of the inner core by an angle $\beta$ towards a longitudinal direction $\phi$ (see Fig. 5a) leads to a change in $\varDelta C_{21}$ and $\varDelta S_{21}$ of (Dumberry 2008a).

22a $$\begin{aligned} \varDelta C_{21}&= - \left( 1+k_2^{r_s} \right) \frac{8\pi }{15} \sqrt{\frac{3}{5}} \frac{r_s^5}{R^2} \frac{\varDelta \rho _{icb}}{M} \epsilon _s \, \sin \beta \, \cos \beta \, \cos \phi \, , \end{aligned}$$

22b $$\begin{aligned} \varDelta S_{21}&= - \left( 1+k_2^{r_s} \right) \frac{8\pi }{15} \sqrt{\frac{3}{5}} \frac{r_s^5}{R^2} \frac{\varDelta \rho _{icb}}{M} \epsilon _s \, \sin \beta \, \cos \beta \, \sin \phi \, . \end{aligned}$$

The Love number $k_2^{r_s}=0.9736$ accounts for the additional contribution to the gravity variation at the surface caused by global elastic deformations. The coefficients of vertical ground deformation at the Earth’s surface associated with this inner core tilt are

23a $$\begin{aligned} \varDelta U_{21}^c&= - h_2^{r_s} \frac{8\pi }{15} \sqrt{\frac{3}{5}} \frac{r_s^5}{R} \frac{\varDelta \rho _{icb}}{M} \epsilon _s \, \sin \beta \, \cos \beta \, \cos \phi \, , \end{aligned}$$

23b $$\begin{aligned} \varDelta U_{21}^s&= - h_2^{r_s} \frac{8\pi }{15} \sqrt{\frac{3}{5}} \frac{r_s^5}{R} \frac{\varDelta \rho _{icb}}{M} \epsilon _s \, \sin \beta \, \cos \beta \, \sin \phi \, . \end{aligned}$$

The Love number $h_2^{r_s}=1.730$ captures the surface displacement associated with the global elastic deformations in response to an inner core tilt. (It was computed in Dumberry (2008a) although its numerical value was not given.)

Assuming that the degree 2, order 2 ICB topography is maximum at longitude equal 0, an axial rotation of a uniform density inner core by an angle $\alpha$ (see Fig. 5b) produces a change in the degree 2, order 2 Stokes coefficients of (e.g., Gillet et al. 2021)

24a $$\begin{aligned} \varDelta C_{22}&= \left( 1+k_2^{r_s} \right) \frac{4\pi }{\sqrt{5}} \frac{r_s^4}{R^2} \frac{\varDelta \rho _{icb}}{M} h_{22}^s \left[ 1 - \cos (2\alpha ) \right] \, , \end{aligned}$$

24b $$\begin{aligned} \varDelta S_{22}&= \left( 1+k_2^{r_s} \right) \frac{4\pi }{\sqrt{5}} \frac{r_s^4}{R^2} \frac{\varDelta \rho _{icb}}{M} h_{22}^s \left[ \sin (2\alpha ) \right] \, , \end{aligned}$$

and a change in the vertical displacement coefficients at the Earth’s surface of

25a $$\begin{aligned} \varDelta U_{22}^c&= h_2^{r_s} \frac{4\pi }{\sqrt{5}} \frac{r_s^4}{R} \frac{\varDelta \rho _{icb}}{M} h_{22}^s \left[ 1 - \cos (2\alpha ) \right] \, , \end{aligned}$$

25b $$\begin{aligned} \varDelta U_{22}^s&= h_2^{r_s} \frac{4\pi }{\sqrt{5}} \frac{r_s^4}{R} \frac{\varDelta \rho _{icb}}{M} h_{22}^s \left[ \sin (2\alpha ) \right] \, . \end{aligned}$$

## Quantitative Estimates of Gravity and Surface Deformation Variations from Core Processes

We now present estimates of the temporal changes in the gravity field and vertical ground deformations from different core processes. To appraise these, it is useful first to give a measure of the observed variations. The amplitude of the decadal changes in $\varDelta C_{20}$ is of the order of $10^{-10}$ whereas that of other Stokes coefficients of degree 2 (orders 1 and 2) are approximately $2-5 \times 10^{-11}$ (Rosat et al. 2021). Interannual variations of degree 2 are closer to $1-2 \times 10^{-11}$ (Rosat et al. 2021). Changes in the Stokes coefficients of degree 3 and 4 are of the same order of magnitude (Sośnica et al. 2015). Because a gravity signal originating from the core should decrease with increasing harmonic degree, this indicates that a large part of the planetary scale, low degree decadal gravity variation is caused by surface processes. Nevertheless, a part of the observed signal may be of core origin and our goal is to determine how large this contribution may be. GPS observations of the ground deformations only cover the past 2 decades and it is more difficult to build accurate models of the decadal changes in the low harmonic degrees. Hence, our focus will be on the gravity field. As an indication, interannual variations of degree 2 are estimated to be of the order of 0.5–1 mm (Rosat et al. 2021).

### Pressure Changes at the Core-Mantle Boundary

At leading order, the force balance in Earth’s core is between the Coriolis acceleration, pressure gradients, the Lorentz force and buoyancy (e.g., Jones 2015),

26 $$\begin{aligned} 2 \rho _o { \varvec{\varOmega } } \times \mathbf{u} = - { \varvec{\nabla } } p + \mathbf{J} \times \mathbf{B} - \rho ' g {\hat{\mathbf{r}}} \, , \end{aligned}$$

where $\mathbf{u}$ is the velocity, $\mathbf{B}$ is the magnetic field, $\mathbf{J}$ is the current density, *g* is the scalar gravitational acceleration, *p* is pressure, $\rho '$ is the density anomaly with respect to the background density $\rho _o$, and ${ \varvec{\varOmega } } = \varOmega _o {\hat{\mathbf{z}}}$ is the Earth’s rotation vector pointing in the axial direction ${\hat{\mathbf{z}}}$. The Lorentz force is expected to be small near the surface of the core (e.g., Bloxham and Jackson 1991; Jault and Le Mouël 1991). Taking ${\hat{\mathbf{r}}} \times$(26), with $u_r=0$, we obtain the condition of tangential geostrophy (Le Mouël 1984; Gire and Le Mouël 1990) at the CMB,

27 $$\begin{aligned} 2 \varOmega _o \rho _f \cos \theta \, \mathbf{u_h} = {\hat{\mathbf{r}}} \times { \varvec{\nabla _h} } p \, , \end{aligned}$$

relating horizontal flows $\mathbf{u_h}=\mathbf{u_h}(\theta ,\phi )$ to horizontal pressure gradients, where ${ \varvec{\nabla _h} }= { \varvec{\nabla } } - {\hat{\mathbf{r}}} \frac{\partial }{\partial r}$ and $\rho _f$ is the density of the fluid at the CMB.

Tangential geostrophy provides a direct connection between core flows and lateral pressure variations at the CMB. Hence, although core flows reconstructed from geomagnetic variations depend on the inversion method and are inherently non-unique, and furthermore that the pressure is likely not perfectly geostrophic, predictions of pressure changes at the CMB based on observations—the temporal changes of the magnetic field observed at the surface—can nevertheless be built.

To our knowledge, the first study relating pressure changes at the CMB to geodetic observables through the elastic mantle deformations that they induce is that of Merriam (1988). His objective was simply to provide an upper bound for the lateral pressure variations at the CMB based on the observed changes in Earth’s rotation and the elliptical gravity coefficient $J_2$ (connected to $C_{20}$ by $J_2 = - \sqrt{5} C_{20}$). The first proper prediction based on tangential geostrophy is from the study of Fang et al. (1996). They calculated the Love numbers ${\bar{k}}_l$ and ${\bar{h}}_l$ that connect pressure at the CMB to gravity changes and surface deformations (see Eqs. 12 and 13) and produced maps of vertical surface displacements at two epochs (1965 and 1975). They showed that core flows can induce ground deformations of the order of 1-3 mm. Their prediction of the gravity variation was restricted to $J_2$, and they showed that the temporal changes in $J_2$ may be as a large as $-1.3 \times 10^{-11}$ $\hbox {yr}^{-1}$, approximately half the observed quasi-linear rate of change that characterizes the rate of $J_2$ change prior to 2000 (e.g., Nerem and Wahr 2011; Sun et al. 2019).

**Fig. 6:**
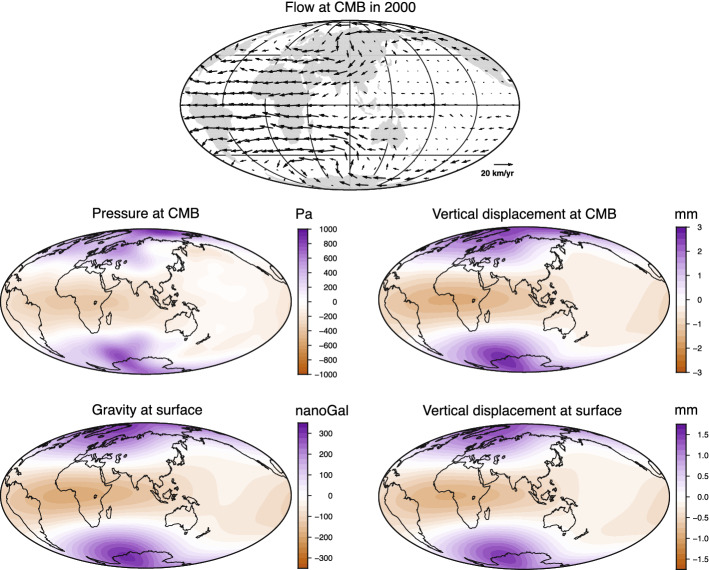
Example of a flow map at the CMB for the year 2000, and its associated geostrophic pressure, vertical displacement at the CMB and surface, and gravity change at the surface. The flow model is the ensemble average of the flow models in Barrois et al. (2017) truncated at degree 11

However, the Love numbers calculated by Fang et al. (1996) failed to account for the deformation of equipotential surfaces within the core. They were recalculated in Dumberry and Bloxham (2004) and in Greff-Lefftz et al. (2004) (see Table 2). We show in Fig. 6 an example of a core flow at the CMB for a single epoch, along with its associated geostrophic pressure map. We also show in the same Figure the vertical deformations at the CMB and surface, and the changes in the gravity field at the surface induced by the CMB pressure.

For a core flow amplitude ${{\mathcal {U}}}$, Eq. (27) gives an estimate of the expected lateral pressure changes at mid-latitude along the CMB of

28 $$\begin{aligned} \varDelta p \sim \sqrt{2} \rho _f \varOmega _o \, {{\mathcal {U}}} L_h \, . \end{aligned}$$

Taking ${{\mathcal {U}}} \sim 5 \times 10^{-4}$ m $\hbox {s}^{-1}$ (approximately 15 km $\hbox {yr}^{-1}$) and a typical horizontal length scale of $L_h \sim 1000$ km gives a typical geostrophic pressure of approximately 500 Pa, consistent with the pressure map shown in Fig. 6. Using Eqs. (4), (5), (12) and (13), this gives a typical large-scale (degree 2) gravity variation at the surface of $\sim 500$ nGal, and vertical deformations at the CMB and surface of $\sim 3$ mm and $\sim 2$ mm, respectively, as observed in Fig. 6. The typical amplitude of the Stokes coefficients of degrees 2, 3 and 4 is $2 \times 10^{-10}$, $5 \times 10^{-11}$ and $2 \times 10^{-11}$, respectively.

A significant portion of core flow circulation at the CMB is quasi-steady (e.g., Bloxham 1992), so the surface deformation of a few mm and gravity variation of a few hundreds of nGal dominantly reflect a stationary contribution from the core. Gravity variations and ground deformations caused by pressure changes at the CMB are connected instead to *changes* in core flows. Typical changes in core flows over a few decades are approximately 1-3 km $\hbox {yr}^{-1}$, an order of magnitude weaker than the quasi-steady part, and taking ${{\mathcal {U}}} \sim 3$ km $\hbox {yr}^{-1}$ $\approx 1 \times 10^{-4}$ m $\hbox {s}^{-1}$, typical pressure variations at the CMB should be $\varDelta p \sim 100$ Pa, and typical gravity and surface deformations should be $\sim 100$ nGal and 0.4 mm, respectively. Changes in Stokes coefficients of degrees 2, 3 and 4 are $4 \times 10^{-11}$, $1 \times 10^{-11}$ and $4 \times 10^{-12}$, respectively. Changes in the coefficients of vertical displacement at the surface of degrees 2, 3 and 4 are 0.2, 0.08 and 0.04 mm, respectively.

**Fig. 7:**
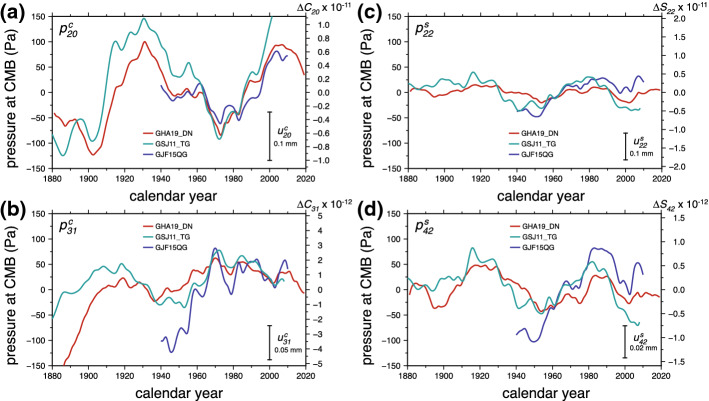
Spherical harmonic coefficients pressure at the CMB as a function of time for different core flow models (see Gillet et al. 2021, for details on flow models. **a** $p_{20}^c$; **b** $p_{22}^s$; **c** $p_{31}^c$; **d**) $p_{42}^s$. On each panel, the change in the associated Stokes coefficient is shown on the right-hand side *y*-axis, and the inset scale shows the change in the associated coefficient of vertical displacement at the surface

Both the studies of Dumberry and Bloxham (2004) and Greff-Lefftz et al. (2004) focused their efforts on the temporal variations of the zonal component of the gravity field. Their primary goal was to determine the contribution from changes in CMB pressure to the variations of $J_2$ about its quasi-linear trend reported by Cox and Chao (2002). Decadal changes in $J_2$ induced by CMB pressure were shown to be of the order of $1 \times 10^{-11}$, consistent with our above estimate, and approximately a factor 10 smaller than the observed fluctuations in $J_2$ (and also, notably, a factor 10 smaller than the earlier prediction made by Fang et al. (1996)). Dumberry (2010) extended these predictions to all Stokes coefficients, zonal and non-zonal, of degrees 2, 3 and 4. The study of Gillet et al. (2021) focused on the changes in vertical deformations at the surface. We show in Fig. 7 a prediction of the temporal variation for a few coefficients of the CMB pressure computed from tangential geostrophy for different core flow models. Typical pressure changes over a few decades are of the order of 50–100 Pa, confirming our above prediction. The temporal variation of the associated Stokes coefficients and coefficients of vertical surface deformations are also shown in Fig. 7 and are also in line with our order of magnitude estimates. The decadal changes in $\varDelta C_{20}$ are approximately $10^{-11}$, 10 times smaller than the observed variations. The changes in other coefficients of degree 2 are of the order of $5 \times 10^{-12}$, a factor 4 to 10 smaller than the observed signals.

For changes at a period of 6 year, typical core flow changes can be as large as those observed over a few decades, ($\sim 3$ km $\hbox {yr}^{-1}$ $\approx 1 \times 10^{-4}$ m $\hbox {s}^{-1}$, e.g., Gillet et al. (2015)) but this is predominantly for smaller scale flows with harmonic degree larger than 4. Sub-decadal core flow changes of low degrees 1–3 are typically a factor 10 weaker ($\sim 0.3$ km $\hbox {yr}^{-1}$ $\approx 1 \times 10^{-5}$ m $\hbox {s}^{-1}$). This implies typical changes in the low harmonic degrees of the CMB pressure, surface gravity and surface deformations of 5 Pa, 5 nGal and 0.02 mm, respectively. Indeed, interannual variations in pressure are clearly discernible in Fig. 7, with amplitudes typically a factor 10 weaker than decadal variations. As shown in Gillet et al. (2021), vertical surface deformations induced by these are of the order of 0.02 mm.

To support their assertion that the 6-year, degree 2, 1 mm signal of vertical ground deformation that they report originates in the core (see Sect. 2.3), Ding and Chao (2018) argue that core flows are expected to produce CMB pressure variations of approximately 500 Pa, which would be sufficient to generate the observed deformation. However, this is only correct for the quasi-steady part of the core flows. At a period of 6 year, the CMB pressure changes and the ground deformation they induce are two orders of magnitude smaller, as demonstrated in Gillet et al. (2021). Likewise, the 6-year variation in the Stokes coefficients $\varDelta C_{22}$ and $\varDelta S_{22}$ of $\sim 1 \times 10^{-11}$ reported in Chao et al. (2020) are a factor 10 larger than the expected magnitude of the signal produced by CMB pressure changes.

The dominant mode of gravity variation reported by Mandea et al. (2012, 2020) (see Sect. 2.2) is of the order of a few hundred nGal with a spatial pattern dominated by spherical harmonic degree $\sim$6. Based on the order of magnitude changes presented above, taking core flow fluctuations of 3 km $\hbox {yr}^{-1}$ $\approx 1 \times 10^{-4}$ m $\hbox {s}^{-1}$, gives surface gravity changes of approximately 100 nGal, but this is for degree 2. Given that ${\bar{k}}_6$ is smaller than ${\bar{k}}_2$ by two orders of magnitude (see Table 2), degree 6 gravity changes driven by CMB pressure should be approximately 1 nGal, two orders of magnitude too small.

That the CMB pressure changes driven by core flows are not sufficiently large to generate a signal of a few hundred nGal was noted by Mandea et al. (2015). They proposed an alternative mechanism, that it is instead sub-decadal changes in CMB topography from the process of dissolution-crystallization that is generating the gravity signal. Because of the density contrast between the mantle and the core, a displacement of the CMB implies a local pressure change. Mandea et al. (2015) argue that this pressure imposed on the core drives a geostrophic flow. The latter feeds a radial flow generating the observed secular acceleration of the vertical component of the magnetic field that is correlated with the gravity signal.

As we have seen, the leading order geostrophic balance (see Eq. 27) indeed implies that a change in core flows leads to a modification of the lateral CMB pressure gradients. But care must be taken when applying the reverse concept, i.e. that a lateral pressure gradient imposed on the core would result in a geostrophic flow. The core should respond to an imposed lateral pressure gradients the same way it responds to an imposed gravity potential (e.g., from tidal origin), that is, primarily by a radial deflection of its equipotential surfaces. The oceans, which are subject to pressure gradients imposed by the atmosphere, offer a good analogy. For (slow) seasonal changes, the principal response of the ocean is a change in sea surface height (the so-called inverted barometer approximation), not the generation of a geostrophic flow (e.g., Gill and Niiler 1973; Ponte et al. 1991).

Hence, although the lateral pressure variations associated with a geostrophic core flow of the order of 10 km $\hbox {yr}^{-1}$ can lead to a CMB deformation of 1 mm (see Fig. 6), the converse is unlikely true, a CMB topography change of 1 mm does not induce a 10 km $\hbox {yr}^{-1}$ geostrophic core flow. If this were the case, large earthquakes, which can entrain a CMB deformation of the order of 1 mm (e.g., Cannelli et al. 2007), would imprint an abrupt reorganization of core flows; no such change is observed in the wake of large earthquakes. At leading order, core flows driven by a CMB topography change should instead result from conservation of potential vorticity, i.e. the change in local vorticity $\zeta$ induced by a change in column height (e.g., Sect. 3.4 of Pedlosky (1987). For a fluid column of original height *H* stretched by $\delta h$, the induced vorticity change is $\zeta \approx 2 \varOmega _o \, \delta h/H$. As an illustration, taking $H= 5000$ km, a change of $\delta h=1$ mm results in a induced vorticity equal to $2.9 \times 10^{-14}$ $\hbox {s}^{-1}$. For a column with a radius of 1000 km, this corresponds to a rotation flow speed of $2.9 \times 10^{-8}$ m $\hbox {s}^{-1}$, or 0.92 m $\hbox {yr}^{-1}$, at its outer edges, 3 orders of magnitude weaker than the observed decadal core flows.

### Density Anomalies in the Fluid Core

An estimate of the typical magnitude of the density anomalies in the fluid core is obtained by taking ${ \varvec{\nabla } } \times$(26) and neglecting Lorentz forces,

29 $$\begin{aligned} - \frac{2 \varOmega _o \rho _o}{g} {\hat{\mathbf{z}}} \cdot { \varvec{\nabla } } \mathbf{u} = {\hat{\mathbf{r}}} \times { \varvec{\nabla _h} } \rho ' \, . \end{aligned}$$

This is the well-known thermal wind balance, connecting axial flow gradients to lateral variations in density. Taking a typical flow amplitude ${{\mathcal {U}}}$ gives a density anomaly estimate of

30 $$\begin{aligned} \rho ' \sim \frac{2 \varOmega _o \rho _o}{g} \frac{L_h}{L_z}\, {{\mathcal {U}}}\, , \end{aligned}$$

where $L_z$ is a typical length scale of the axial shear of ${{\mathcal {U}}}$ and $L_h$ is a typical length scale of the horizontal gradient in $\rho '$. Taking $g \sim 10$ m $\hbox {s}^{-2}$, $\rho _o \sim 10^4$ kg $\hbox {m}^{-3}$, ${{\mathcal {U}}} \sim 5 \times 10^{-4}$ m $\hbox {s}^{-1}$, $L_h \sim L_z$, gives a typical density anomaly of $\rho ' \sim 7 \times 10^{-5}$ kg $\hbox {m}^{-3}$ in the fluid core (Stevenson 1987). This estimate is consistent with that based on the heat flow carried by convective fluid motions (Jones 2015).

Integrated over the thickness of the core in Eq. (9), this gives Stokes coefficients of degrees 2, 3 and 4 approximately equal to $10^{-10}$, $4 \times 10^{-11}$ and $10^{-11}$, respectively. These estimates are appropriate for the quasi-steady part of the flow. For a flow amplitude of ${{\mathcal {U}}} \sim 1 \times 10^{-4}$ m $\hbox {s}^{-1}$ more typical of the decadal and interannual variations, these estimates are decreased by a factor 5.

These are similar to the estimates that we obtained from pressure variations at the CMB. Hence, while mass anomalies in the core are very small, they can nevertheless contribute to a significant gravity variation. The order of magnitude given above is instructive, but it represents an upper bound because it assumes that core flows are restricted to thermal winds, purely driven by density variations. This is certainly not the case. In fact, we expect geostrophic flows—flows with no gradients in the direction of the rotation axis—to dominate the dynamics at interannual and decadal timescales (Jault 2008), in which case the gravity changes produced by density anomalies should be smaller.

The main difficulty in building a prediction of gravity variations from $\rho '$ is that its variations everywhere in the core cannot be reconstructed solely based on core flows at the CMB. Possible dynamical scenarios were suggested in Dumberry (2010) in order to do this, although ultimately they remain based a scaling between density and core flows similar to that of Eq. (30). Predictions of the gravity signal from density anomalies were computed in Dumberry (2010) and shown to be smaller though of the same order of magnitude as the contribution from pressure at the CMB, consistent with our simple estimates above. Furthermore, Dumberry (2010) showed that the gravity signal from pressure and density tend to be anti-correlated. Physically, this is because regions of low (high) density in the core should correspond to upwellings (downwellings) and to regions of high (low) pressure at the CMB. Thus, areas of negative (positive) gravity anomalies from low (high) density should coincide with areas of positive (negative) gravity anomalies caused by the uplift (depression) of the CMB and associated elastic deformations.

Though instructive, predictions of the gravity signal from density anomalies in the core such as those computed in Dumberry (2010) remain unconstrained. If decadal core flows reflect primarily the dynamics of axially invariant geostrophic motions, the density-induced gravity variations should be smaller than the pressure-induced part, although it may not be negligible. To shed some light on this issue, one option is to use numerical models of the geodynamo. To date, the only study to have computed the gravity signal in a dynamo model is that of Jiang et al. (2007) and it is restricted to the gravity anomalies at the CMB, not the Earth’s surface. They find that the pressure contribution dominates for axisymmetric harmonics, but that the contributions from density and pressure are approximately equally important for the non-axisymmetric harmonics and have a tendency to be anti-correlated. Care has to be taken when scaling the results of such simulations to Earth. Nevertheless, these results are broadly consistent with those suggested in Dumberry (2010).

### Radial Motion in a Stably Stratified Layer

An alternative way to generate density variations within the volume of the core is through radial motion in a stably stratified layer. For a layer characterized by a sub-adiabatic density gradient of

31 $$\begin{aligned} \frac{\partial \rho _o}{\partial r} = - N^2 \frac{\rho _o}{g} \, , \end{aligned}$$

where *N* is the buoyancy frequency, a radial displacement $u_r$ leads to a density anomaly

32 $$\begin{aligned} \rho ' = u_r \frac{\partial \rho _o}{\partial r} = - N^2 \frac{u_r \rho _o}{g}\, . \end{aligned}$$

A stably stratified layer at the top of the core has been suggested on the basis of seismological observations (e.g., Tanaka 2007; Helffrich and Kaneshima 2010), either caused by a thermal (e.g., Pozzo et al. 2012; Labrosse 2015) or compositional (e.g., Fearn and Loper 1981; Buffett and Seagle 2010; Gubbins and Davies 2013) stratification.

An example of such radial motion is that associated with zonal Magnetic Archimedes Coriolis (MAC) waves, which possibly account for the decadal zonal flows at the CMB (Buffett 2014; Buffett and Knezek 2018). With a layer 140 km thick, and assuming *N* is linearly increasing from zero at the base of the layer to a maximum approximately equal to the Earth rotation frequency $\varOmega _o$ at the top, zonal MAC waves have periods of a few decades. This gives a mean sub-adiabatic density gradient within the layer of $\sim - 2.7 \times 10^{-6}$ kg $\hbox {m}^{-4}$. For the mode of oscillation with harmonic degree 4 which has a period close to 60 year, the radial velocity within the layer is approximately a factor 100 smaller than the azimuthal velocity ${{\mathcal {U}}}$ (see Fig. 2 of Buffett (2014). Taking ${{\mathcal {U}}} \sim 1 \times 10^{-4}$ m $\hbox {s}^{-1}$ as a typical zonal flow over a period of 60 year gives a radial displacement of approximately $u_r \sim 300$ m. Combined with our estimate of the sub-adiabatic gradient, this gives a density variation of $\rho ' \sim 8 \times 10^{-4}$ kg $\hbox {m}^{-3}$. This is a factor 10 larger than the density changes from convection that we estimated in the previous subsection. However, these anomalies are restricted to the thin stratified layer, less than one tenth the thickness of the core, so the estimate of the gravity variation computed from Eq. (9) would be smaller than that estimated in the previous section. In addition, because the form of zonal MAC waves involves a radial gradient, positive mass anomalies would be partly cancelled by negative mass anomalies resulting in a weaker net gravity variation.

Although the above estimate for $\rho '$ is obtained for a specific MAC wave, it provides an adequate measure of the expected amplitude of $\rho '$ within a layer of the same thickness. This can be appreciated by the thermal wind balance of Eq. (30), which still applies (provided we neglect Lorentz forces) although here $\rho '$ is instead due to radial motion in a stratified layer and given by Eq. (32). In other words, the maximum density anomaly in a stratified layer remains bound by the magnitude of the horizontal flow. Taking $L_z$ 10 times smaller than $L_h$, to mimic motion trapped in a stratified layer, increases the estimate of $\rho '$ from the thermal wind equation by a factor 10, consistent with our estimate based on the zonal MAC wave above. If the sub-adiabatic density gradient is increased, the radial displacement is decreased by an inversely proportional amount such that $\rho '$ remains unchanged. If the layer thickness is reduced, $\rho '$ can be larger, but the radial integration in Eq. (9) is over a thinner layer and the gravity prediction would not change substantially. Hence, whether from excited natural modes or dynamically forced by convection underneath, radial motions in a stratified layer at the top of the core do not produce gravity variations that are larger than those estimated from pressure at the CMB in Sect. 4.1.

### Longitudinal Reorientation of the Inner Core

The gravity signal caused by changes in the longitudinal orientation of the long equatorial axis of the inner core—denoted by an angle $\alpha$—is determined by Eq. (24). We do not have direct observations of the time-history of $\alpha$, so a precise prediction of this gravity signal is not possible. However, estimates of its amplitude can be built from three different lines of reasoning.

First, $\alpha$ can be inferred from the observed time-varying zonal flows ($v_{\phi }$) inside the tangent cylinder at the CMB (e.g., Gillet et al. 2021). Assuming that the time-varying zonal flows near the ICB are similar in magnitude as those near the CMB, and further assuming that electromagnetic coupling at the ICB is sufficiently strong to entrain the inner core (e.g., Gubbins 1981), then a mean zonal flow $v_\phi$ inside the tangent cylinder oscillating at frequency $\omega$ is connected to $\alpha$ by $v_{\phi } = r_s \omega \alpha$. At a period of 30 year ($\omega = 2\pi /30$ $\hbox {yr}^{-1}$), azimuthal flows in the vicinity of the tangent cylinder at the core surface are approximately 2 km $\hbox {yr}^{-1}$ (e.g., Gillet et al. 2015), which corresponds to an oscillation amplitude of $\alpha \approx 8 \times 10^{-3}$ rad, or $0.4^\circ$.

A second estimate is obtained from the observed $\varDelta$LOD. Changes in $\alpha$ induce a gravitational torque on the mantle (e.g., Buffett 1996a, b). Assuming that the resulting $\varDelta$LOD are entirely due to this gravitational torque, the orientation of the inner core topography is determined by (Buffett and Creager 1999)

33 $$\begin{aligned} \alpha = - \frac{2\pi }{T_o^2} \frac{C_m}{\varGamma } \frac{d}{dt} \, \varDelta LOD \, , \end{aligned}$$

where $T_o=86400$ s is Earth’s rotation period, $C_m$ is the polar moment of inertia of the mantle and $\varGamma$ is a constant that captures the amplitude of the torque. The latest estimate of $\varGamma$ ranges from $3\times 10^{19}$ to $2\times 10^{20}$ N m (Davies et al. 2014). Taking a rate of change of $\varDelta$LOD equal to 2 ms over 10 years (approximately the largest rate of $\varDelta$LOD observed in the past 100 year) gives a range of $\alpha$ between $0.1^\circ$ and $0.7^\circ$ for the high and low choice of $\varGamma$, respectively. This is similar and consistent with our first estimate of $\alpha$.

A third estimate can be built from seismic rays passing through the inner core for repeating earthquakes that have similar waveforms, the so-called earthquake doublets (e.g., Zhang et al. 2005). If the temporal shift of the waveforms is interpreted as a change in inner core rotation speed, Tkalcic et al. (2013) show that fluctuations in inner core rotation may be as large as $0.5^\circ$ yr $^{-1}$ with a typical period of 20 year, giving an amplitude of $\alpha = 0.5^{\circ }/\text{yr } \cdot (20 \text{ yr } / 2\pi ) \approx 1.5^\circ$.

Each of these lines of reasoning has its own caveats. In particular, the first and third of these are estimates of the fluctuations in the axial rotation of the bulk of the inner core. If the characteristic timescale of viscous deformation within the inner core is of the same order or shorter than the fluctuation period, the ICB topography relaxes back toward its equilibrium alignment with the mantle. The viscosity of the inner core is not well known, but inferences from high-pressure experiments (e.g., Gleason and Mao 2013), first-principle calculations (e.g., Ritterbex and Tsuchiya 2020) and geodynamics (e.g., Buffett 1997; Greff-Lefftz et al. 2000; Koot and Dumberry 2011; Davies et al. 2014) all point to a relatively weak inner core which deforms significantly over a few years. The estimate of $\alpha$ from methods 1 and 3 is then an upper bound and $\alpha$ may actually be much smaller.

Nevertheless, taking the largest of these estimates, $\alpha =1.5^\circ$, using the parameters of Table 1, and $h_{22}^s=18$ m (based on a degree 2, order 2 geoid at the CMB of $h_{22}^f=50$ m and assuming the ICB is an equipotential surface) in Eq. (24) gives decadal fluctuations of $\varDelta S_{22} \approx 5 \times 10^{-11}$, similar to the observed fluctuations of $S_{22}$ over the past few decades (e.g., Rosat et al. 2021). The amplitude of the vertical ground deformation at the Earth’s surface from Eq. (25) gives $\varDelta U_{22}^s \approx 0.3$ mm. Using a more conservative estimate of $\alpha =0.5^\circ$ reduces these predictions of $\varDelta S_{22}$ and $U_{22}^s$ by a factor 3. If a significant amount of viscous relaxation takes place within the inner core, these predictions would be further reduced. However, $h_{22}^s$ may be larger than our estimate of 18 m if the geoid at the CMB is larger than $h_{22}^f=50$ m and/or if $h_{22}^s$ includes an additional non-hydrostatic contribution.

While the above estimates give hope that longitudinal inner core fluctuations may be detectable geodetically, they also illustrate that the parameters on which this prediction depends are not well constrained (notably $\alpha$, $h_{22}^s$ and the inner core viscosity). The fact that the observed decadal changes in $\varDelta S_{22}$ are not substantially different in amplitude than those of other low degree coefficients suggests that it is highly doubtful that the observed $\varDelta S_{22}$ changes are solely caused by fluctuations in inner core rotation. Yet, our simple order of magnitude estimate indicates that the inner core may contribute a part to the observed signal.

Focusing on a period of 6 year, taking a typical zonal flow amplitude of $v_\phi =0.4$ km $\hbox {yr}^{-1}$ inside the tangent cylinder (e.g., Gillet et al. 2015, 2019), the first line of reasoning to estimate $\alpha$ gives $\alpha \approx 3 \times 10^{-4}$ rad, or $0.018^\circ$. This yields $\varDelta S_{22} \approx 6 \times 10^{-13}$ and $U_{22}^s \approx 0.04$ mm, smaller by factors of 30 and 5, respectively, compared to the amplitude of the observed signals (Rosat et al. 2021). The amplitude of the 6-year $\varDelta$LOD oscillation is $\sim 0.1$ ms (Holme and de Viron 2013), and based on the second line of reasoning, a higher amplitude of $\alpha =0.06^\circ$ to $0.4^\circ$ is allowed. Taking the largest of these, $\alpha =0.4^\circ$, gives $\varDelta S_{22} \approx 10^{-11}$ and $U_{22}^s \approx 0.1$ mm, approximately their observed amplitudes. However, inner core fluctuations of $\alpha =0.4^\circ$ at a period of 6 year would require zonal flows near the ICB that are 20 times larger than those observed at the CMB at interannual periods, which is doubtful.

### Meridional Reorientation of the Inner Core

An equatorial rotation of the inner core (a tilt of its elliptical figure) induces gravity variations of degree 2, order 1. If the time-dependent variations in the angle of tilt ($\beta$) and its longitudinal orientation ($\phi$) are known, a prediction of $\varDelta C_{21}$ and $\varDelta S_{21}$ can be built from Eq. (22). Although plausible scenarios have been proposed for the time-dependent equatorial torque that may be applied on the inner core (e.g., Dumberry and Bloxham 2002; Mound 2005; Dumberry 2008b), it is not possible to build a prediction of $\beta$ and $\phi$ based on geomagnetic observations alone.

Proceeding in reverse, the inner core tilt angle that is required to produce the observed decadal changes in $\varDelta C_{21}$ and $\varDelta S_{21}$ of $\sim 5\times 10^{-11}$ is $\beta \approx 0.07^\circ$. Because of the large inertial and gravitational torque resisting an inner core tilt, a large equatorial torque of the order of $10^{20}$ N m must be applied on the inner core in order generate a such a tilt (e.g., Dumberry and Bloxham 2002; Dumberry 2008b). To put this amplitude in perspective, this is two orders of magnitude larger than the torque in the axial direction applied by the core on the mantle in order to explain the observed decadal $\varDelta$LOD. If significant viscous deformations take place within the inner core on a decadal timescale, the figure of the inner core relaxes back towards an alignment with the mantle, requiring an even larger torque in order to achieve the same angle of tilt. Although it cannot be ruled out that changes in inner core tilt could contribute to the observed decadal changes in $\varDelta C_{21}$ and $\varDelta S_{21}$, in all likelihood, it represents a minor or negligible contribution.

At interannual periods, the observed changes in $\varDelta C_{21}$ and $\varDelta S_{21}$ of the order of $10^{-11}$ require smaller fluctuations in inner core tilt of $\beta \approx 0.014^\circ$. However, since the required periodic torque must be delivered over a shorter time period, its amplitude is not substantially different than $10^{20}$ N m. It is difficult to imagine a dynamical scenario that can produce such a large torque. This implies that, in all likelihood, changes in inner core tilt only contribute to a small or negligible fraction of the observed changes in $\varDelta C_{21}$ and $\varDelta S_{21}$ at interannual periods.

### Polar Motion

If driven by mass redistribution, polar motion—the displacement of the position where the rotation axis intersects the Earth’s surface—captures the changing orientation of the rotation vector as it tracks the changing moment of inertia tensor of the planet. Denoting the two orthogonal polar motion components by $m_1$ and $m_2$, with $m_1$ aligned with the Greenwich meridian, they are connected to the Stokes coefficients of degree 2, order 1 (e.g., Gross 2015) by

34 $$\begin{aligned} m_1 = \sqrt{\frac{5}{3}} \frac{M R^2}{A(e-\kappa )} \varDelta C_{21} \, , \quad m_2 = \sqrt{\frac{5}{3}} \frac{M R^2}{A(e-\kappa )} \varDelta S_{21} \, , \end{aligned}$$

where $A=8.0115 \times 10^{37}$ kg $\hbox {m}^2$ is the mean equatorial moment of inertia of the whole Earth, $e=3.247 \times 10^{-3}$ is the dynamical ellipticity and the factor $\kappa = 1.039 \times 10^{-3}$ accounts for elastic deformations associated with the change in centrifugal potential induced by the polar motion (the numerical values of *A*, *e* and $\kappa$ are taken from Mathews et al. 1991). Equation (34) assumes that none of the fluid regions of Earth (core or fluid layers at the surface) carry angular momentum in the equatorial direction and that the mass redistribution is internal (in other words, that it does not load the Earth).

Polar motion offers an additional way to monitor large-scale global mass redistributions with a possible contribution from the core. The decadal polar motion is of the order of 10–25 milliarcsec (mas) (e.g., Gross 2015). Since the early 2000s, when satellite gravity observations have allowed to monitor the planetary scale changes in terrestrial water storage more accurately, the latter have been shown to account for most of the non-steady drift in polar motion (e.g., Adhikari and Ivins 2016). This suggests that they probably also account for most of the earlier decadal variations. Nevertheless, in the interest of investigating a possible contribution from the core, based on Eq. (34), a polar motion of 10 mas requires a $\varDelta C_{21}$, $\varDelta S_{21}$ of approximately $2.5 \times 10^{-11}$. There is also a polar motion signal with a period of 5.9 year with an amplitude of $\sim$3 mas after the known effects from surface processes have been removed (Chen et al. 2019); if caused by an internal mass redistribution, this requires $\varDelta C_{21}$, $\varDelta S_{21}$ $\approx 7.5 \times 10^{-12}$.

As an indication, in order to produce a polar motion of 1 mas, a CMB pressure change of degree 2, order 1 of approximately 16 Pa is required. With our previous estimate of decadal fluctuations of degree 2 pressure changes, approximately 50 Pa, this indicates that CMB pressure changes could generate a decadal polar motion signal of 2-3 mas. This is a factor 10 smaller than the observed signal. Likewise, CMB pressure changes of the order of 5 Pa at a period of 6 year would lead to a polar motion signal of 0.3 mas, also a factor 10 smaller than the observed signal. Note, however, that changes in $p_{21}^c$ and $p_{21}^s$ require core flows that are anti-symmetric with respect to the equatorial plane. Since decadal and sub-decadal fluctuations in core flows are expected to be dominantly symmetric, changes in $p_{21}^c$ and $p_{21}^s$ can be significantly smaller. In fact, in core flow models where equatorial symmetry is enforced (e.g., Gillet et al. 2011), $p_{21}^c$ and $p_{21}^s$ are identically zero.

An inner core tilt is another way to generate a degree 2, order 1 internal mass redistribution. As indicated in the previous subsection, a $\varDelta C_{21}$, $\varDelta S_{21}$ change of $5\times 10^{-11}$ (leading to a polar motion of 20 mas) requires an inner core tilt of $0.07^\circ$. A polar motion of 1 mas would require an inner core tilt of $0.0035^\circ$ (which would produce a $\varDelta C_{21}$, $\varDelta S_{21}$ change of $2.5\times 10^{-12}$). The amplitude of the required torque is approximately $10^{19}$ N m; if a decadal torque of that magnitude can be generated (e.g., Dumberry 2008b), changes in inner core tilt may contribute to a fraction of the observed polar motion, both at decadal and interannual timescales.

## Discussion and Conclusions

A summary of the estimates of the changes in the degree 2 Stokes coefficients from the different core processes is presented in Table 3, along with their observed amplitudes. Likewise, a summary of the changes in the degree 2 coefficients of the ground deformations are presented in Table 4. Observations of the decadal variations in ground deformation are not very accurate given the limited timespan of the GPS records and we have left them blank. The values listed for the contribution from density anomalies are based on the results of Dumberry (2010) where they were shown to be typically half the size of the contribution from the CMB pressure. However, we emphasize that these values are highly uncertain and could be lower. The gravity signal from the inner core rotation is based on the amplitude of the longitudinal misalignment $\alpha$ estimated from the zonal core flows inside the tangent cylinder at the CMB. The values listed for the inner core tilt signal are not a prediction, but are instead an indication based on an inner core tilt that would lead to decadal and 6-year polar motions of 1 and 0.1 mas, respectively.

**Table 3:** Observed Stokes coefficients (no units) of gravity variations of degree 2 and contribution from core processes

	$\varDelta C_{20}$		$\varDelta C_{21}$, $\varDelta S_{21}$		$\varDelta C_{22}$, $\varDelta S_{22}$	
	Decadal	6 year	Decadal	6 year	Decadal	6 year
Observed	1	0.5	0.5	0.2	0.5	0.2
CMB pressure	0.1	0.01	0.05	0.005	0.05	0.005
Density $\hbox {anomalies}^\dagger$	0.05	0.005	0.025	0.0025	0.025	0.0025
Inner core rotation					0.2	0.006
Inner core $\hbox {tilt}^\ddagger$			0.025	0.0025		

All numerical values are multiplied by a factor $10^{-10}$. To retrieve amplitudes in units of nGal, numerical values for $m=0$, 1 and 2 should be multiplied by $6.59\times 10^{12}$ (for the amplitude at the poles), $1.14 \times 10^{13}$ and $5.70 \times 10^{12}$, respectively

^†^Set to half of the contribution from CMB pressure based on the results in Dumberry (2010)

^‡^Not a prediction, but assuming an inner core tilt generating a decadal (6 year) polar motion of 1 (0.1) mas

**Table 4:** Observed spherical harmonic coefficients of ground deformations of degree 2 and contribution from core processes

	$\varDelta U^c_{20}$		$\varDelta U^c_{21}$, $\varDelta U^s_{21}$		$\varDelta U^c_{22}$, $\varDelta U^s_{22}$	
	Decadal	6 year	Decadal	6 year	Decadal	6 year
Observed	–	0.75	–	0.5	–	0.2
CMB pressure	0.2	0.02	0.2	0.02	0.2	0.02
Density $\hbox {anomalies}^\dagger$	0.1	0.01	0.1	0.01	0.1	0.01
Inner core rotation					0.1	0.04
Inner core $\hbox {tilt}^\ddagger$			0.014	0.0014		

All numerical values are in mm

^†^Set to half of the contribution from CMB pressure based on the results in Dumberry (2010).

^‡^Not a prediction, but assuming an inner core tilt generating a decadal (6 year) polar motion of 1 (0.1) mas

The largest predicted decadal gravity signal originating from the core is that caused by axial fluctuations in inner core rotation, involving a change in $\varDelta S_{22}$ of the order of $2\times 10^{-11}$, which is of the same order of magnitude as the observed signal. We stress again that this is an upper bound, assuming no viscous relaxation of the inner core. If significant viscous relaxation occurs over a few years, the associated gravity signal is reduced. Other gravity signals of core origin are typically a factor 10 smaller than the observed signals, both at a decadal timescale and at a period of 6 year. Likewise, the predicted amplitudes of ground deformations at sub-decadal periods are typically a factor 10 smaller than the observed changes.

Based on our present-day understanding of dynamical processes in the core, the redistribution of mass and ground deformation they can generate appear too weak to explain the amplitude of the observed decadal and sub-decadal signals. If indeed correct, the implication is that the observed variations must be predominantly driven by surface processes. Based on the numbers given in Tables 3 and 4, core processes may contribute to a fraction of the observed variations, perhaps as much as 10%. This offers hope that some of these processes may eventually be detectable geodetically. Of course, this will only be possible provided that the dominant contributions from surface processes are adequately modelled and removed. This remains a challenge at present, but undoubtedly models of these processes will continue to improve in the coming years.

This conclusion though leaves important questions unanswered, namely, why then should there be any temporal correlations between gravity and magnetic field variations, as reported by Mandea et al. (2012, 2020), or between ground deformations and magnetic and LOD variations, as demonstrated in Ding and Chao (2018). These correlations may point to a dynamical link between core dynamics and surface processes that we have yet to understand. From this viewpoint, the survey of core processes—and the magnitude of the signals they can generate—that we have presented in these pages may be incomplete, simply reflecting a milepost in our evolving understanding.

This review has focused on the ways by which core dynamics can drive variations in gravity and ground deformation at the Earth’s surface. The reversed dynamical link, whether a mass redistribution at the Earth’s surface can drive core flows, remains an unexplored territory. This can occur either directly via the change in the imposed gravitational potential, but also indirectly, for instance by a change in mantle rotation driven by surface processes.

As we alluded to at the end of Sect. 4.1, the primary response of the fluid core to an imposed change in gravitational potential should be a radial deflection of its equipotential surfaces, not the excitation of core flows. The radial deflections of the CMB and ICB from this, which can be of the order of 1 mm, can induce vorticity by stretching or compressing fluid columns in the axial direction. The simple order of magnitude given in Sect. 4.1 though suggests that flows generated in this manner are of the order of 1 m $\hbox {yr}^{-1}$, 3 orders of magnitude weaker than typical decadal core flows.

However, the inner core being solid, equipotential surfaces within the latter cannot fully adjust to an altered potential. A change in potential of degree 2 may then lead to a rotation of the inner core so that its degree 2 density structure realigns with the perturbed potential. Because we expect a strong electromagnetic coupling at the ICB, an inner core rotation drives core flows. We can build a simple order of magnitude estimate for such flows. Let us focus on an axial change in the rotation rate of the inner core, $\varOmega _s$. The latter is driven by a gravitational torque $\varGamma _z$ according to

35 $$\begin{aligned} C_s \frac{d \varOmega _s}{dt} = \varGamma _z \, , \end{aligned}$$

where the polar moment of inertia of the inner core is $C_s = 5.83 \times 10^{34}$ kg $\hbox {m}^3$ (e.g., Mathews et al. 1991). The torque $\varGamma _z$ can be computed as the following integral over the volume of the inner core (e.g., Dumberry 2008b, Eq.A1)

36 $$\begin{aligned} \varGamma _z = - {\hat{\mathbf{z}}} \cdot \int _V \big ( \rho _s - \rho _{fs} \big ) \mathbf{r} \times {\varvec{\nabla }} \delta \varPhi \, dV \, , \end{aligned}$$

where $\rho _s$ is the density within the inner core and $\rho _{fs}$ is the density of the fluid core at the ICB. Taking the long axis of the inner core at rest to be aligned with the Greenwich meridian, the gravitational potential change $\delta \varPhi$ associated with a mass redistribution at the surface driving an inner core rotation is $\delta \varPhi = (r/R)^l (GM/R) \varDelta S_{lm} Y_l^m$, where $Y_l^m$ is the surface spherical harmonic. The largest contribution to $\varGamma _z$ is from the product between the degree 2, order 2 ICB topography $h_{22}^s$ and $\varDelta S_{22}$, and an order of magnitude estimate of $\varGamma _z$ is given by

37 $$\begin{aligned} \varGamma _z \approx h_{22}^s \, \varDelta S_{22} \, \varDelta \rho _{icb} \, G M \frac{{r_s}^4}{R^3}\, . \end{aligned}$$

Taking values for $\varDelta \rho _{icb}$, *G*, *M*, $r_s$ and *R* from Table 1, $h_{22}^s \approx 18$ m (see Sect. 4.4) and a typical change of $\varDelta S_{22} \approx 5 \times 10^{-11}$ over decades gives $\varGamma _z \approx 1.8 \times 10^{12}$ N m. Such a torque drives a change in $\varOmega _s$ of $1.8 \times 10^{-5}$ deg $\hbox {yr}^{-1}$ over a ten-year period, corresponding to a lateral speed at the ICB of 0.38 m $\hbox {yr}^{-1}$. This is smaller than the typical decadal core flow changes by more than 3 orders of magnitude. Although crude, this simple estimate suggests that core flows driven by an inner core rotation in response to a mass redistribution at the Earth’s surface is a negligible contribution to core dynamics.

For a tilt of the elliptical inner core, the stretching and compressing of fluid columns in the axial direction inside the tangent cylinder represent an additional source of core flows. For a tilt by an angle $\beta$, the change in the ICB topography at mid-latitude is approximately $2 \beta r_s \epsilon _s$, where $r_s$ is the inner core radius and $\epsilon _s \approx 2.5 \times 10^{-3}$ is the geometrical ellipticity. The amplitude of the polar motion (approximately 20 mas at decadal periods) gives a measure of the equivalent tilt angle of the mantle produce by a degree 2, order 1 surface mass change. A tilt of the inner core by the same angle ($\beta = 20$ mas $\approx 1 \times 10^{-7}$ rad), so that it is completely realigned with the mantle, corresponds to a mid-latitude topography change of 0.3 mm, generating flows of the order of 0.3 m $\hbox {yr}^{-1}$, a negligible contribution to core dynamics.

A change in mantle rotation (axial or equatorial) induced by surface processes entrains flows in the core by virtue of coupling at the CMB and also by gravitational coupling with the inner core (a change in the rotation of the latter can then entrain core flows by coupling at the ICB). These core flows induce a secondary change in mantle rotation through conservation of angular momentum. For a perfect coupling (i.e. a rigid rotation of the core tracking the mantle), because the moment of inertia of the core is smaller than that of the mantle, the additional change in mantle rotation would be approximately a factor 10 smaller than the original perturbation driven by surface processes. Given that the coupling at the CMB is far from perfect at decadal and shorter timescales, the speed of core flows entrained by a change in mantle rotation is necessarily much weaker than the change in mantle speed. The feedback that the core may have on a mantle rotation change induced by surface processes should then be very limited, but this simple line of reasoning ignores possible amplification mechanisms. Can core flows forced by a change in mantle rotation lead to observable changes in the magnetic field? It is a question that may be worth exploring in further details.

Further investigating ways in which core dynamics or CMB processes can generate global mass redistribution and deformation, and ways in which these may be connected to dynamical processes in the atmosphere, oceans and hydrosphere, will ultimately improve our understanding of Earth’s global dynamics. In this spirit, highlighting further correlations between geomagnetic and geodetic signals can further help to point us in the right direction.

## Footnotes

1 http://www-app2.gfz-potsdam.de/pb1/op/champ/.

2 https://earth.esa.int/eogateway/missions/swarm.

3 http://www.csr.utexas.edu/grace/.

4 https://www.nasa.gov/missions/grace-fo.

5 http://www.ftp.legos.obs-mip.fr/pub/soa/gravimetry/grace_legos.

6 http://www.spacecenter.dk/files/magnetic-models/CHAOS-6/.
